# Development and Validation of a Novel Ferroptosis-Related Gene Signature for Prognosis and Immunotherapy in Hepatocellular Carcinoma

**DOI:** 10.3389/fmolb.2022.940575

**Published:** 2022-06-30

**Authors:** Bo Zhang, Jilong Zhao, Bing Liu, Yanan Shang, Fei Chen, Sidi Zhang, Jiayao He, Yumei Fan, Ke Tan

**Affiliations:** ^1^ Key Laboratory of Molecular and Cellular Biology of Ministry of Education, College of Life Sciences, Hebei Normal University, Shijiazhuang, China; ^2^ Key Laboratory of Animal Physiology, Biochemistry and Molecular Biology of Hebei Province, College of Life Sciences, Hebei Normal University, Shijiazhuang, China; ^3^ Department of Neurosurgery, Handan Central Hospital, Hebei Medical University, Shijiazhuang, China

**Keywords:** ferroptosis, hepatocellular carcinoma, prognosis, FRG signature, immune infiltration

## Abstract

Hepatocellular carcinoma (HCC) is a cancer that is sensitive to ferroptosis, and immunotherapy has emerged as a promising treatment for HCC patients. However, the prognostic potential of ferroptosis-related genes (FRGs) and the effect of ferroptosis on the tumor immune microenvironment in HCC remain largely obscure. Here, we analyzed the expression pattern of FRGs using the TCGA, ICGC and GEO databases. The expression of most FRGs was upregulated in HCC tissues compared with normal liver tissues. Three independent clusters were determined by consensus clustering analysis based on FRG expression in HCC. Cluster 3 exhibited higher expression, unfavorable prognosis, and higher histological tumor stage and grade than clusters 1 and 2. CIBERSORT analysis indicated different infiltrating levels of various immune cells among the three clusters. Moreover, most immune checkpoint genes were highly expressed in cluster 3. Univariate Cox regression and LASSO regression analyses were performed to develop a five FRG-based prognostic risk model using the TCGA and ICGC datasets. Kaplan–Meier analysis and ROC curves were performed to verify the prognostic potential of the risk model. A nomogram containing independent prognostic factors was further developed. Compared with low-risk patients, high-risk HCC patients exhibited worse overall survival (OS). In addition, this risk model was significantly correlated with the infiltrating levels of six major types of immune cells in HCC. Finally, the relationships between the five FRGs and drug sensitivity were investigated. The present study suggests that the five FRGs could elucidate the molecular mechanisms of HCC and lead to a new direction for the improvement of predictive, preventive, and personalized medicine for HCC.

## Introduction

Primary liver cancer is a global, pathological and fatal malignancy, with approximately 906,000 new liver cancer cases and 830,000 deaths according to the statistics of GLOBOCAN in 2020 (Sung et al., 2021). Liver cancer is the sixth most frequently diagnosed cancer and ranks as the third leading cause of cancer-related death in the world. Hepatocellular carcinoma (HCC) is the most common histological type of primary liver cancer, accounting for approximately 90% of cases (Forner et al., 2018). Over the decades, great progress has been achieved in multidisciplinary treatments, including surgery, liver transplantation, chemotherapy, radiotherapy, monoclonal antibody therapy and immunotherapy. However, the overall survival (OS) rate of HCC patients remains to be further improved, especially for advanced-stage HCC patients (Forner et al., 2018; Sung et al., 2021). Therefore, it is urgent to discover more effective prognostic models and therapeutic targets for HCC. The rapid development of bioinformatics has undoubtedly provided important methods and platforms for screening prognostic biomarkers in cancer patients.

Successful avoidance of programmed cell death, such as apoptosis, necrosis and autophagic cell death, is a major common feature of cancer. The discovery of a new form of regulatory cell death called ferroptosis, provides new therapeutic targets and strategies for human tumors (Dixon et al., 2012). The occurrence of ferroptosis depends on the lethal accumulation of cellular iron, the overwhelming generation of reactive oxygen species (ROS), lipid peroxidation and the unbalanced oxidative stress response (Chen J. et al., 2022). As research continues to expand and deepen, an increasing number of genes and signaling pathways that regulate ferroptosis are gradually being discovered (Chen X. et al., 2021; Li et al., 2021; Zhang et al., 2021). Accumulating evidence implies that ferroptosis is closely associated with the development and progression of diverse human cancers, including HCC (Liu et al., 2021; Lei et al., 2022). Sorafenib, a multikinase inhibitor, is the first-line drug for HCC patients that induces apoptosis, inhibits cell proliferation, and suppresses angiogenesis. Interestingly, recent studies have found that sorafenib could induce ferroptotic cell death in HCC, which adds a piece to the puzzle of its anticancer mechanisms (Gao et al., 2021; Wang Q. et al., 2021). In addition to sorafenib, an increasing number of small molecules or drugs have been identified to induce ferroptosis through different targets or signaling pathways in HCC (Nie et al., 2018). Many genes have been identified as suppressors or drivers in the development of HCC and of ferroptosis in HCC. NRF2 protects HCC cells from sorafenib- or erastin-induced ferroptosis by upregulating the expression of different antioxidants, such as NQO1, HO-1 and FTH1 (Sun et al., 2016b). Mechanistically, erastin or sorafenib treatment inhibited the degradation of NRF2, increased the nuclear translocation of NRF2 and facilitated the interaction between NRF2 and its transcriptional coactivator (Sun et al., 2016b). Metallothionein-1G is a suppressor of ferroptosis in HCC and could serve as a promising therapeutic target in sorafenib-resistant HCC cells (Sun et al., 2016a). The increased expression of metallothionein-1G could mediate GSH depletion and lipid peroxidation without affecting the intracellular iron concentrations. CDGSH iron sulfur domain 1 (CISD1) negatively modulates ferroptosis by limiting mitochondrial iron uptake and alleviating lipid peroxidation (Yuan et al., 2016). ACSL4 was identified as an important determinant of ferroptosis sensitivity (Doll et al., 2017). The ACSL4 protein level was significantly and negatively correlated with the half-maximal inhibitory concentration (IC50) value of sorafenib in a panel of HCC cell lines (Feng et al., 2021). Silencing ACSL4 inhibited sorafenib-induced lipid peroxidation and ferroptotic cell death in HCC cells, indicating that ACSL4 may be a potential predictive biomarker of HCC. Moreover, ETS proto-oncogene 1 (EST1)-mediated upregulation of miR-23a-3p suppressed ACSL4 transcription by directly binding to the 3′-UTR of ASCL4 (Lu et al., 2022). Inhibition of miR-23a-3p triggered ferroptosis by rescuing the expression of ASCL4 in sorafenib-treated cells (Lu et al., 2022). Glycolytic enzyme enolase 1 (ENO1), acting as an RNA-binding protein, facilitates the mRNA degradation of IRP1 to mediate iron homeostasis, inhibits the expression of mitoferrin-1 (Mfrn1) and suppresses ferroptosis in HCC cells, suggesting that the ENO1-IRP1-Mfrn1 axis regulates the pathogenesis of HCC and ferroptotic cell death (Zhang et al., 2022). Copper metabolism MURR1 domain 10 (COMMD10) suppressed HIF1α degradation and the nuclear translocation of HIF1α, thus inhibiting the transcription of ceruloplasmin and SLC7A11 to increase ferroptosis and radiosensitivity by disrupting Cu-Fe homeostasis in HCC (Yang et al., 2022). Additionally, CRISPR-based loss-of-function genetic screens identified phosphoseryl-tRNA kinase (PSTK) as a regulator of ferroptosis by inactivating GPX4 and disrupting glutathione metabolism in HCC (Chen Y. et al., 2022). Nevertheless, the relationships between ferroptosis-associated genes and the prognosis of HCC patients still need to be deeply explored.

The present study systematically examined the differential expression of ferroptosis-related genes (FRGs) in HCC and investigated the relationships between the FRG signature and the prognosis and tumor microenvironment (TME) of HCC patients. Clustering subgroups and a five-FRG model were established to optimize prognostic risk stratification. Moreover, the clinical features, risk score, immune cell infiltration, immune microenvironment and drug sensitivity of the five FRGs were thoroughly analyzed to deeply evaluate the effects of the FRGs in HCC. In conclusion, this study attempts to explore the prognostic power of FRGs and immunotherapy strategies for patients with HCC.

## Materials and Methods

### Data Collection and Processing

The RNA sequencing (RNA-seq) data (level 3) and corresponding clinical information of HCC patients were obtained from The Cancer Genome Atlas (TCGA) database (https://portal.gdc.cancer.gov), including 371 HCC cancer samples and 50 normal samples. The RNA-seq data of 226 normal human liver samples were downloaded from the Genotype-Tissue Expression (GTEx) database. Moreover, the RNA-seq data and clinical information from the external validation cohort were extracted from the International Cancer Gene Consortium (ICGC) database (https://dcc.icgc.org/releases/current/Projects, including 240 HCC samples and 202 normal samples), GSE36376 (240 HCC samples and 193 normal liver samples) and GSE10143 (80 HCC samples and 307 normal liver samples) datasets.

### Analysis of the Expression of Ferroptosis-Related Genes

A total of 25 FRGs were obtained from a previous study to systematically analyze the aberrances and functional implications of ferroptosis in HCC (Liu et al., 2020). Differential gene expression analysis was performed by comparing HCC tissues and normal liver tissues using the R packages ggplot2 and pheatmap in the TCGA and ICGC datasets. The Wilcoxon test was used to assess the significance between two groups.

### Immunohistochemistry Analysis

The protein levels in HCC tissues and normal liver tissues from the online Human Protein Atlas (HPA) database (https://www.proteinatlas.org/) were obtained based on immunohistochemical (IHC) staining results.

### Analysis of the Protein–Protein Interaction Network

With the aim of analyzing the Protein–Protein Interaction (PPI) network between different genes, the STRING (https://string-db.org/) online platform was used to evaluate the interactions among FRGs in HCC (Fan et al., 2021). The Metascape online platform was used in the construction of a visual PPI network. Subsequently, Gene Ontology (GO) and Kyoto Encyclopedia of Genes and Genomes (KEGG) enrichment analyses of FRGs were performed using Metascape.

### Ferroptosis-Related Gene-Based Consensus Clustering Analysis

The number of unsupervised clusters and their stability in the TCGA and IGCG datasets were estimated based on the mRNA expression profiles of 25 FRGs with the consensus clustering method using the ConsensusClusterPlus package (v1.54.0). The maximum number of clusters was 6, and 80% of the total samples were drawn 100 times. The R software package pheatmap (v1.0.12) was used for clustering heatmaps. The R (v4.0.3) packages survival and survminer were used for the Kaplan–Meier survival analyses of different subtypes.

### Construction and Validation of a Prognostic Ferroptosis-Related Gene Signature

Univariate Cox analysis of the OS of HCC patients was first carried out to identify FRGs with prognostic potential in HCC with the threshold of *p* < 0.01. A prognostic risk signature of 5 differential FRGs was established by using least absolute shrinkage and selection operator (LASSO) regression analysis in the TCGA training and ICGC validation cohorts. The risk scores of HCC patients were calculated according to the normalized expression level of each FRG and its corresponding regression coefficient. The equation was established as follows: risk score = sum of coefficients × prognostic FRGs expression levels. According to this equation, the risk score of each patient was separately calculated in the TCGA training and ICGC validation cohorts. Subsequently, the patients were stratified into high- and low-risk groups, and the median value of the risk score was set as the cutoff point.

The predictive ability of the nomogram and other clinical parameters (age, sex, TNM stage and grade) for the 1-, 3- and 5-year OS of patients was investigated. Calibration curves were carried out to illustrate the uniformity between the actual outcome and the predicted outcome of the model.

### Evaluation of Immune Cell Infiltration Levels in Hepatocellular Carcinoma

Immune cell infiltration scores were calculated based on the CIBERSORT and TIMER algorithms and were compared between the two risk groups. The infiltration levels of different immune cells in each HCC sample were investigated using the R package immunedeconv. The results of the heatmap were exhibited by the R package pheatmap. The Kruskal–Wallis test was used to assess the significance of the three groups. In addition, the expression of the immune checkpoint genes in three subgroups was examined using the R packages ggplot2 and pheatmap. The expression differences of the key immune checkpoint molecules, including PD-1, CTLA-4 and PD-L1, in the high- and low-risk groups were further analyzed using the R packages ggplot2 and pheatmap. The Wilcoxon test was used to assess the significance between two groups.

### Prediction of the Immunotherapeutic Response

The Tumor Immune Dysfunction and Exclusion (TIDE) score was used to predict the potential clinical immunotherapy response of HCC patients using the R (v4.0.3) packages ggplot2 and ggpubr. TIDE is an algorithm for predicting the clinical response to immune checkpoint inhibitors using TCGA RNA-seq data and the corresponding clinical information of HCC patients.

### Genomics of Drug Sensitivity in Cancer Database

Based on Genomics of Drug Sensitivity in Cancer (GDSC) 2016 (www.cancerrxgene.org/), the chemotherapeutic sensitivity according to the IC50 value of each HCC sample was estimated using the R package pRRophetic. We investigated the different sensitivities of common liver cancer chemotherapy drugs between the high-expression group and the low-expression group.

### Statistical Analysis

All bioinformatic statistical analyses of the present study were performed in R (v4.0.3) software. Differences between the high- and low-risk groups were compared through the Wilcoxon test. Differences in three different groups were compared through the Kruskal–Wallis test. Kaplan–Meier curve analysis was performed by the log-rank test to compare the survival of HCC patients. Pearson correlation analysis was also performed to calculate the correlation coefficient between two variables.

## Results

### Expression of Ferroptosis-Related Genes in Hepatocellular Carcinoma in the The Cancer Genome Atlas Database

**FIGURE 1:**
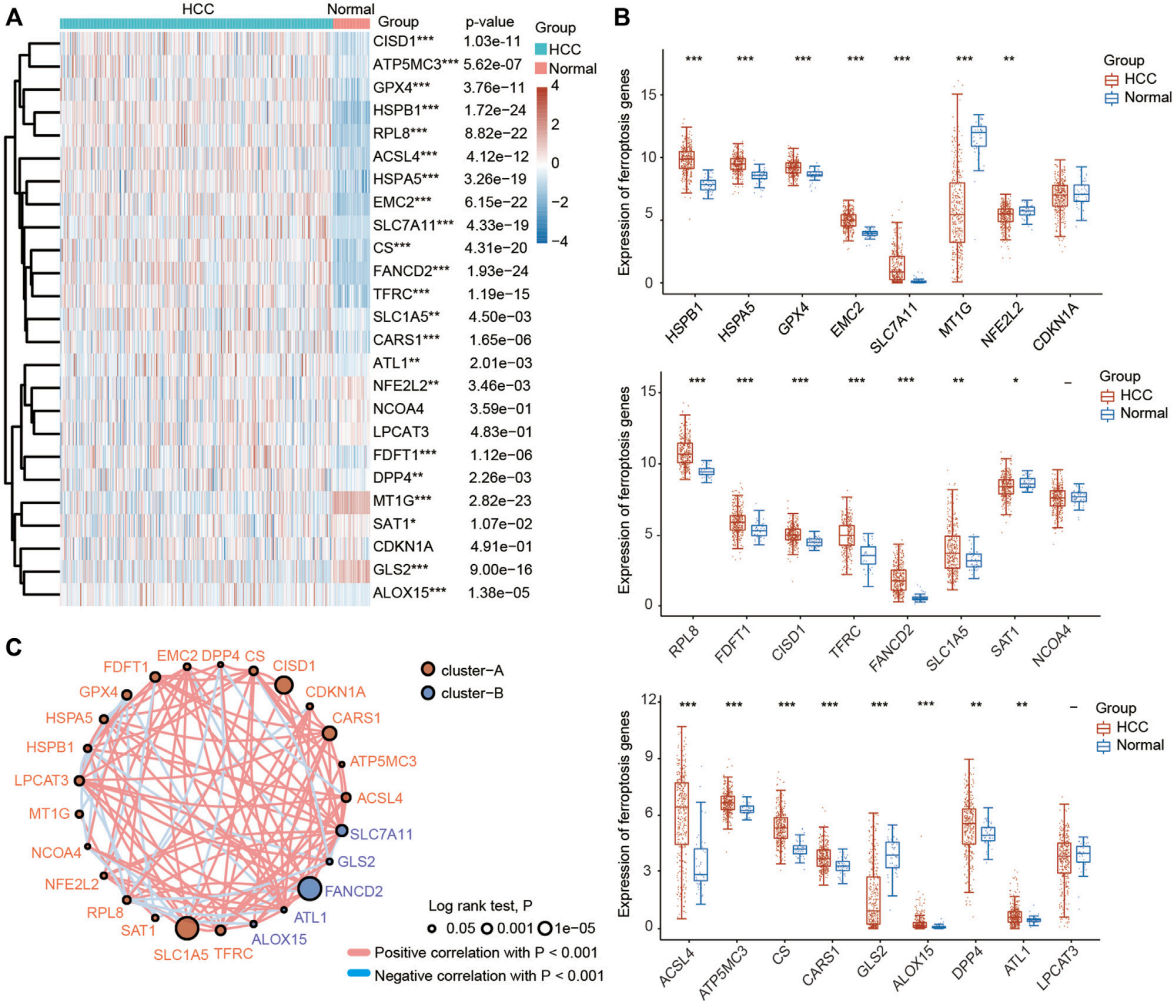
Most ferroptosis-related genes (FRGs) were upregulated in HCC patients based on the TCGA database. **(A)** Heatmap showing the differential expression of 25 FRGs in normal individuals and HCC patients. Blue represents tumor tissues and red represents the normal tissues. The difference in gene expression was compared statistically with the Wilcoxon test. **(B)** Violin plots showing the differential expression of 25 FRGs in normal individuals and HCC patients. Red represents tumor tissues and blue represents the normal tissues. The interquartile range of values was represented by the upper and lower ends in the box and the median value was represented by the lines in the boxes. The difference in gene expression was compared statistically with the Wilcoxon test. **(C)** Correlation and prognostic values of FRGs in HCC. The interactions between genes were shown by linking lines and the correlation strength between genes was shown by thickness. The effect of each FRG on the prognosis was indicated by the size of the circle and Log-rank test. The red and blue lines represent positive and negative correlations, respectively. *p < 0.05, **p < 0.01, ***p < 0.001.

A previous study identified twenty-four genes that play important roles in modulating ferroptotic cell death (Liu et al., 2020). To explore the function of ferroptosis in HCC, we first investigated the differential expression of these ferroptosis regulator genes in HCC tissues and normal liver tissues using the TCGA database. As shown in Figures 1A,B, the expression of HSPA5, EMC2, SLC7A11, HSPB1, GPX4, FANCD2, CISD1, FDFT1, SLC1A5, TFRC, RPL8, DPP4, CS, CARS1, ATP5MC3, ALOX15, ACSL4 and ATL1 was significantly increased in HCC tissues compared with normal liver tissues. In contrast, the expression of NFE2L2, MT1G, SAT1 and GLS2 was greatly decreased in HCC tissues compared with normal liver tissues. In addition, the results of the correlation analysis combined with gene expression and prognosis indicated that most FRGs were positively associated with HCC and exhibited prognostic value in HCC (Figure 1C).

### Validation of the Changed Expression of Ferroptosis-Related Genes in the International Cancer Gene Consortium Database

To confirm the results obtained from the TCGA database, we further evaluated the expression levels of these FRGs using the ICGC database. Consistent with the results obtained from the TCGA database, we found that the expression levels of HSPA5, EMC2, SLC7A11, HSPB1, GPX4, FANCD2, CISD1, FDFT1, SLC1A5, TFRC, RPL8, LPCAT3, DPP4, CS, CARS1, ATP5MC3, ALOX15, ACSL4 and ATL1 were higher in HCC tissues than in normal liver tissues, while the expression levels of CDKN1A, NFE2L2, MT1G, SAT1 and GLS2 were obviously lower in HCC tissues than in normal liver tissues (Supplementary Figures S1A,B). Moreover, the expression of most ferroptosis regulators was positively correlated with prognosis in HCC according to the ICGC database (Supplementary Figure S1C). In addition, the differential expression of these FRGs was further confirmed by the GSE10143 and GSE36376 datasets (Supplementary Figures 2A,B).

### Construction of an Interactive Network of Ferroptosis-Related Genes and Correlation Analysis

**FIGURE 2:**
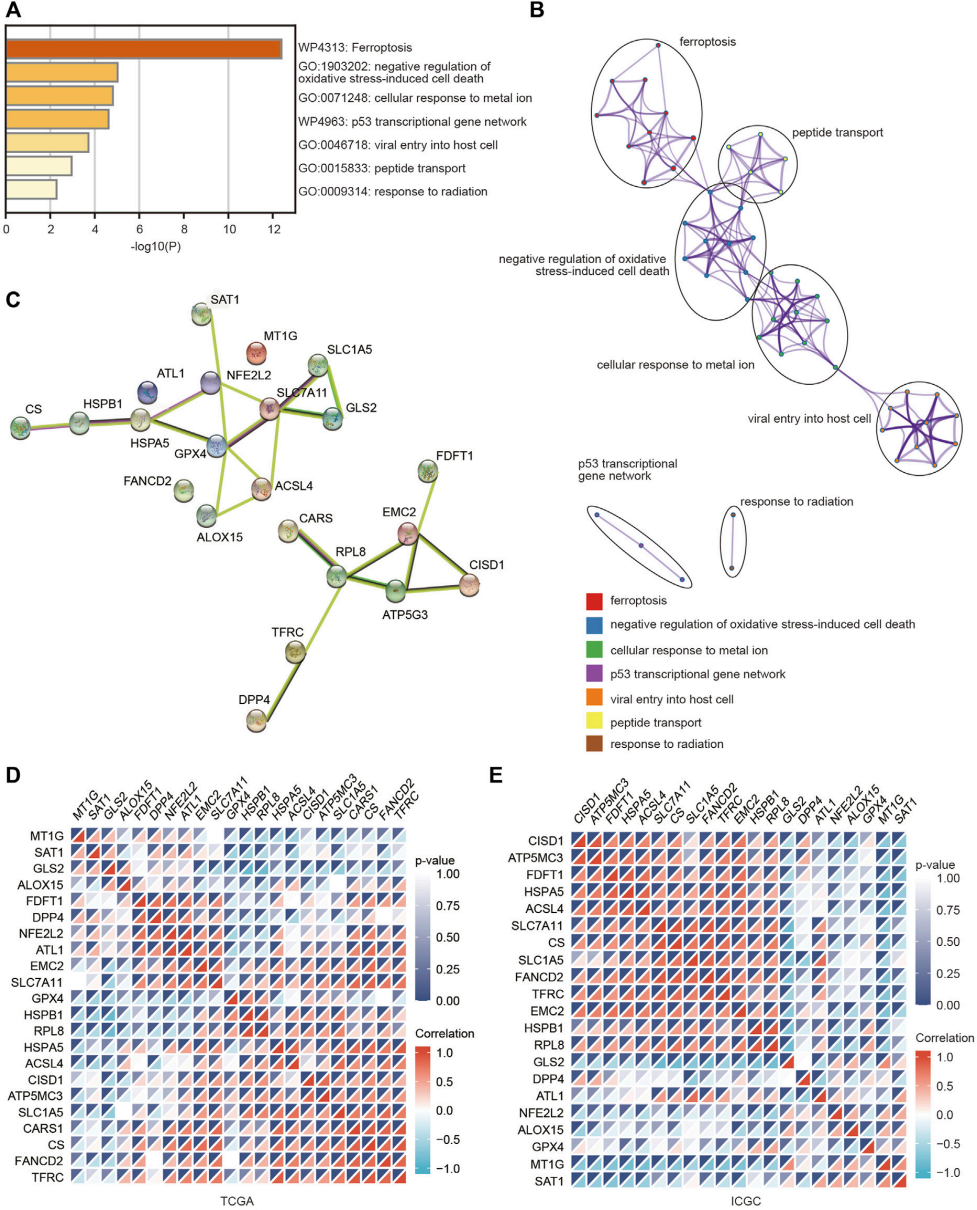
Pathway and functional enrichment analyses of FRGs were performed to explore the role of ferroptosis in HCC. **(A)** Bar graph summary of FRG-enriched pathways. **(B)** A PPI network was obtained from the Metascape database. **(C)** A PPI network was obtained from the STRING database. **(D,E)** Heatmaps showing the correlations among these FRGs in the TCGA and ICGC databases using the spearman analysis. Red represents a positive correlation and blue represents a negative correlation.

To deeply explore the molecular function of these FRGs with changed expression in HCC, pathway and process enrichment analyses were performed based on the Metascape online tool. As shown in Figure 2A, these genes were significantly associated with ferroptosis, negative regulation of oxidative stress-induced cell death, cellular response to metal ions, p53 transcriptional gene network, viral entry into host cells, peptide transport and response to radiation. Furthermore, the PPI network was constructed using the Metascape and STRING online databases. The protein interaction was relatively strong, and various types of interactions are shown in Figures 2B,C. We then assessed the correlations among the FRGs based on the TCGA and ICGC databases, and significant positive or negative correlations between these FRGs were observed (Figures 2D,E).

### Consensus Clustering Analysis of Ferroptosis-Related Genes in Hepatocellular Carcinoma

**FIGURE 3:**
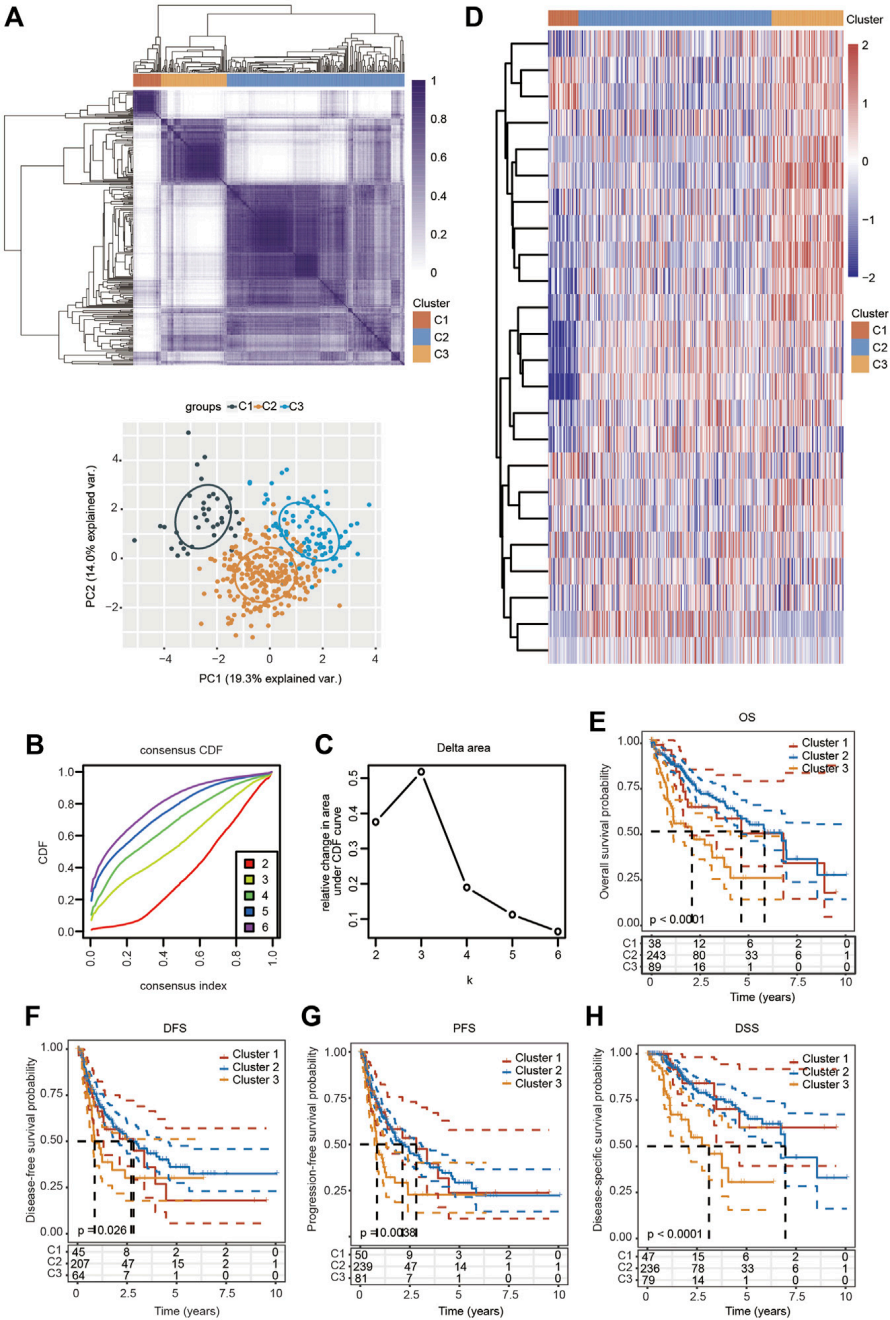
Survival analysis and changed expression of FRGs in distinct HCC clusters in the TCGA cohort. **(A)** Consensus clustering matrix for *k* = 3. **(B,C)** Cumulative distribution function curves for *k* = 2–6. **(D)** Heatmap visualizing the expression patterns of FRGs in three clusters. **(E–H)** The Kaplan–Meier curves indicate the OS **(E)**, DFS **(F)**, PFS **(G)** and DSS **(H)** for three clusters of HCC patients.

Based on the distinct expression level of FRGs and the proportion of ambiguous clustering measures, consensus clustering analysis was performed, and *k* (clustering variable) was increased from 2 to 6. We identified *k* = 3 as the optimal clustering stability, suggesting that it was suitable to separate HCC patients into three subgroups (Figure 3A). Principal component analysis (PCA) according to the whole transcriptome profiles of the 3 clusters was conducted (Figures 3A–C). Compared with cluster 1 and cluster 2, most FRGs were highly expressed in cluster 3 (Figure 3D). Additionally, HCC patients in cluster 3 exhibited worse OS, disease-free survival (DFS), progression-free survival (PFS) and disease-specific survival (DSS) than those in cluster 1 and cluster 2 (Figures 3E–H). Subsequently, significant differences were obtained in different characteristic groups, including T stage, TNM stage and grade groups, among the three clusters (Table 1).

**TABLE 1:** Correlations between different clinicopathological parameters and the three clusters in HCC. The p-values are shown in bold.

	Characteristic	C1	C2	C3	*p*_value
Status	Alive	33	167	41	
	Dead	17	72	41	**0.005**
Age	Mean (SD)	58.9 (12.8)	60 (13.7)	58.2 (13.3)	
	Median [Min, Max]	60 [24, 80]	62 [16, 90]	60 [20, 81]	**0.343**
Sex	Female	12	81	28	
	Male	38	158	54	**0.377**
Race	American Indian	1		1	
	Asian	24	93	41	
	Black	1	13	3	
	White	20	128	36	**0.243**
pT-stage	T1	28	129	24	
	T2	16	52	24	
	T3	2	27	16	
	T3a	2	17	10	
	T3b	1	2	3	
	T4	1	8	4	
	T2b		1		
	TX		1		
	T2a			1	**0.007**
pN-stage	N0	34	162	56	
	NX	16	75	23	
	N1		2	2	**0.906**
pM-stage	M0	37	167	62	
	M1	1	3		
	MX	12	69	20	**0.613**
pTNM-stage	I	28	120	23	
	II	16	49	21	
	IIIA	3	37	25	
	IIIB	1	4	3	
	IIIC	1	4	4	
	IV	1	1		
	III		3		
	IVA		1		
	IVB		2		**0.001**
Grade	G1	7	44	4	
	G2	27	119	31	
	G3	13	69	40	
	G4	3	3	6	**0**

### Association of Patient Clusters With Immune Cell Infiltration in Hepatocellular Carcinoma

**FIGURE 4:**
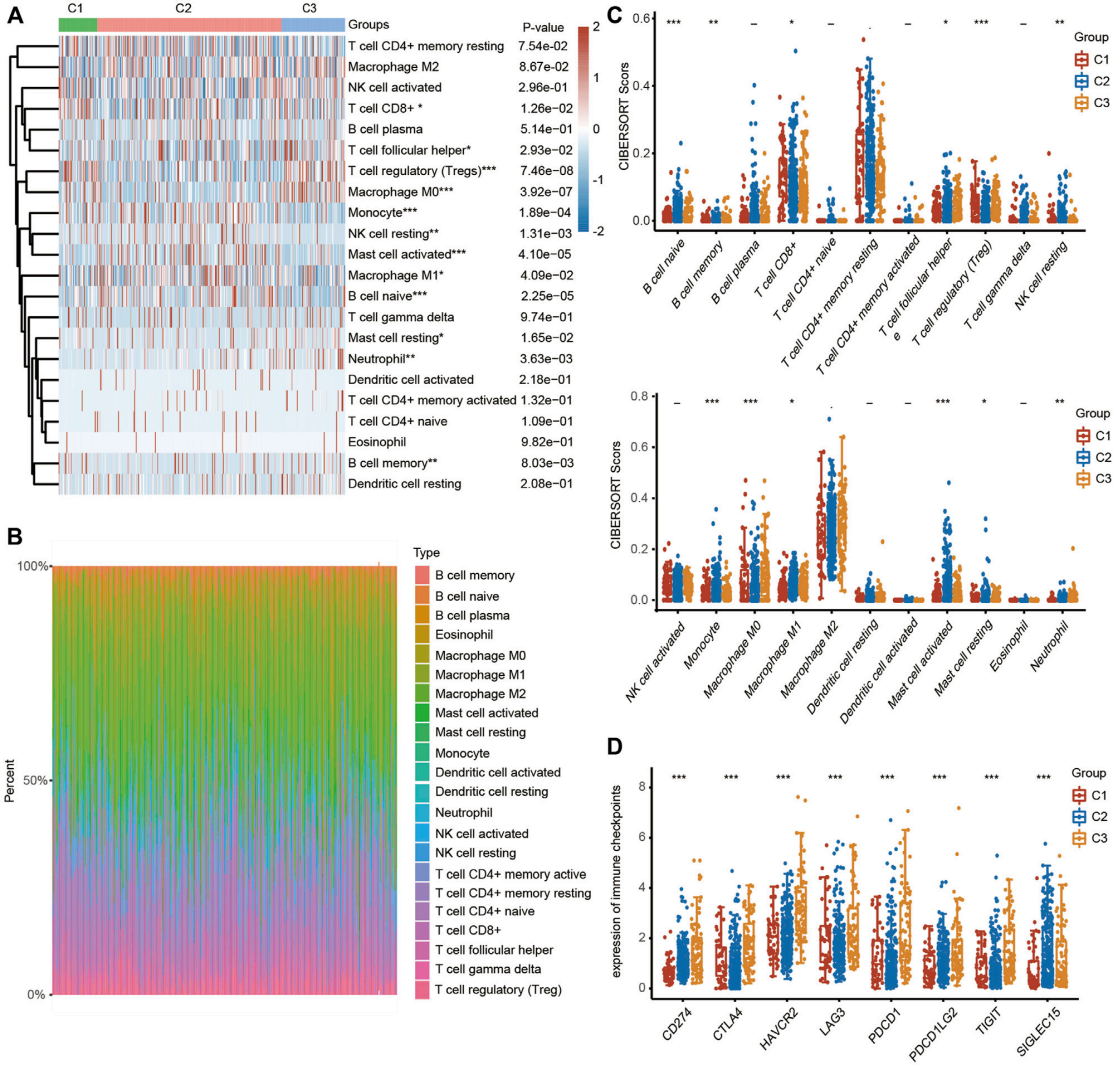
TME characteristics in three different HCC clusters. (FRGs in three clusters are positively correlated with immune cell infiltration in HCC). **(A)** Heatmap showing the different infiltration levels of immune cells in the three clusters. **(B)** Bar plot showing the composition of different immune cells in each patient from the three clusters analyzed by CIBERSORT. **(C)** Violin plots showing the different infiltration levels of immune cells in the three clusters. The difference in three clusters was compared statistically with the Kruskal–Wallis test. **(D)** Violin plots showing the different expression levels of immune checkpoint genes in the three clusters. The difference in gene expression was compared statistically with the Kruskal–Wallis test. The interquartile range of values is represented by the upper and lower ends in the box and the median value is represented by the lines in the boxes. The outliers are shown as dots. *p < 0.05, **p < 0.01, ***p < 0.001.

To explore the correlation between ferroptosis and the tumor immune microenvironment (TAM), we examined the effect of FRGs on immune cell infiltration in HCC. The heatmap results demonstrated that the infiltration scores of naïve B cells, memory B cells, CD8+ T cells, follicular helper T cells (Tfh cells), regulatory T cells (Tregs), resting NK cells, monocytes, M0 macrophages, M1 macrophages, activated mast cells, resting mast cells and neutrophils were significantly different among the C1, C2 and C3 clusters using the CIBERSORT algorithm (Figure 4A). The percentage abundances of tumor-infiltrating immune cells in each sample, with different colors and different types of immune cells using the CIBERSORT algorithm, are shown in Figure 4B. We further evaluated the impact of FRGs on the expression of several important immune checkpoints. Higher expression of CD274 (PD-1), CTLA-4, HAVCR2, LAG3, PDCD-1 (PD-L1), PDCD1LG2 and TIGIT was found in cluster 3 than in cluster 1 and cluster 2, while the highest expression of SIGLCE15 was observed in the cluster 2 (Figure 4C).

We also examined the expression of the 24 FRGs among these three clusters and found that the expression of CDKN1A, HSPA5, EMC2, SLC7A11, NFEL2L, HSPB1, GPX4, FANCD2, CISD1, FDFT1, SLC1A5, TFRC, RPL8, NCOA4, LPCAT3, GLS2, DPP4, CS, CARS1, ATP5MC3, ALOX15, ACSL4 and ATL1 was significantly different in these three clusters (Supplementary Figure S3A). Additionally, we examined the correlation between clusters and the response to immunotherapy to further explore the clinical application value of the model. HCC patients in cluster 3 had higher TIDE scores, suggesting a higher possibility of immune escape (Supplementary Figure S3B).

### Identification of Prognostic Ferroptosis-Related Genes in the TCGA-HCC Cohort

**FIGURE 5:**
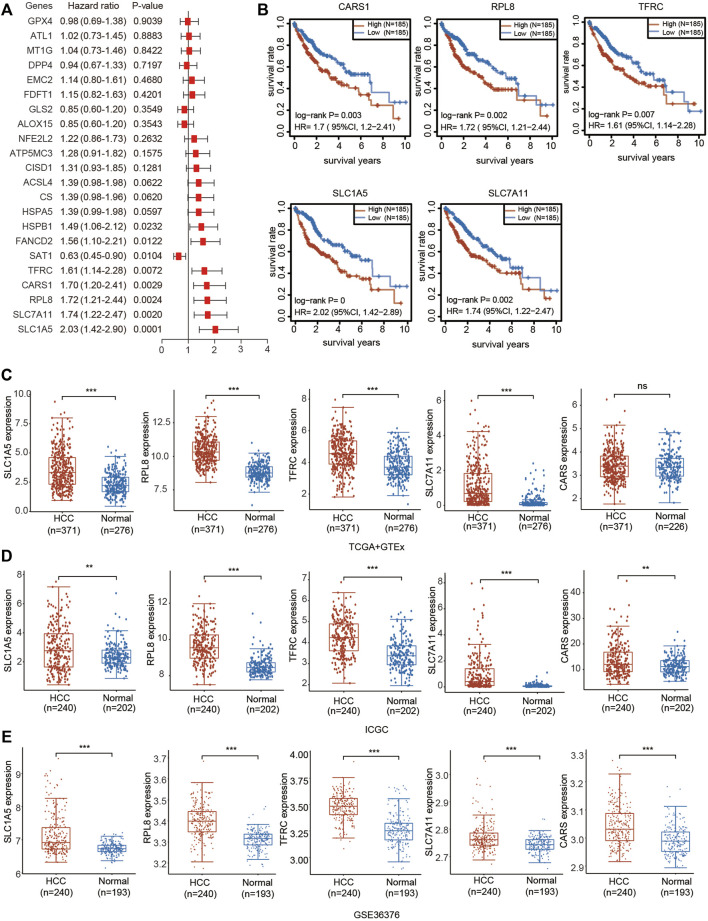
Prognostic value and upregulated expression of five FRGs. **(A)** Univariate Cox regression analysis of the prognostic potential of FRGs in HCC. **(B)** The Kaplan–Meier curves indicate the prognostic values of CARS1, RPL8, TFRC, SLC1A5 and SLC7A11 (*p* < 0.05, Log-rank test). **(C)** Upregulated expression of CARS1, RPL8, TFRC, SLC1A5 and SLC7A11 in the TCGA combined with GTEx databases. The difference in gene expression was compared statistically with the Wilcoxon test. **(D)** Upregulated expression of CARS1, RPL8, TFRC, SLC1A5 and SLC7A11 in the ICGC databases. The difference in gene expression was compared statistically with the Wilcoxon test. **(E)** Upregulated expression of CARS1, RPL8, TFRC, SLC1A5 and SLC7A11 in GSE36376. The difference in gene expression was compared statistically with the Wilcoxon test. *p < 0.05, **p < 0.01, ***p < 0.001.

We then performed univariate Cox regression analysis of the differentially expressed genes in Figure 1 to select prognostic FRGs in HCC. Five genes were selected from the univariate Cox regression analysis with a cutoff of *p* < 0.01, including SLC7A11, SLC1A5, TFRC, RPL8 and CARS1 (Figure 5A). The Kaplan–Meier analysis results further indicated that the increased expression of SLC7A11, SLC1A5, TFRC, RPL8 and CARS1 corresponded with unfavorable OS in HCC patients (Figure 5B). Thus, these five genes were selected as hub genes for further analysis.

The expression of these five genes was investigated based on the TCGA, ICGC and Gene Expression Omnibus (GEO) databases. Consistent with the results obtained from TCGA (Figure 1), upregulated expression of SLC7A11, SLC1A5, TFRC, RPL8 and CARS1 was observed in HCC tissues compared with adjacent normal liver tissues according to the TCGA + GTEx and ICGC databases (Figures 5C,D). SLC7A11, SLC1A5, TFRC, RPL8 and CARS1 were also highly expressed in HCC tissues according to the GSE10143 and GSE36376 datasets (Figure 5E; Supplementary Figure S4).

**FIGURE 6:**
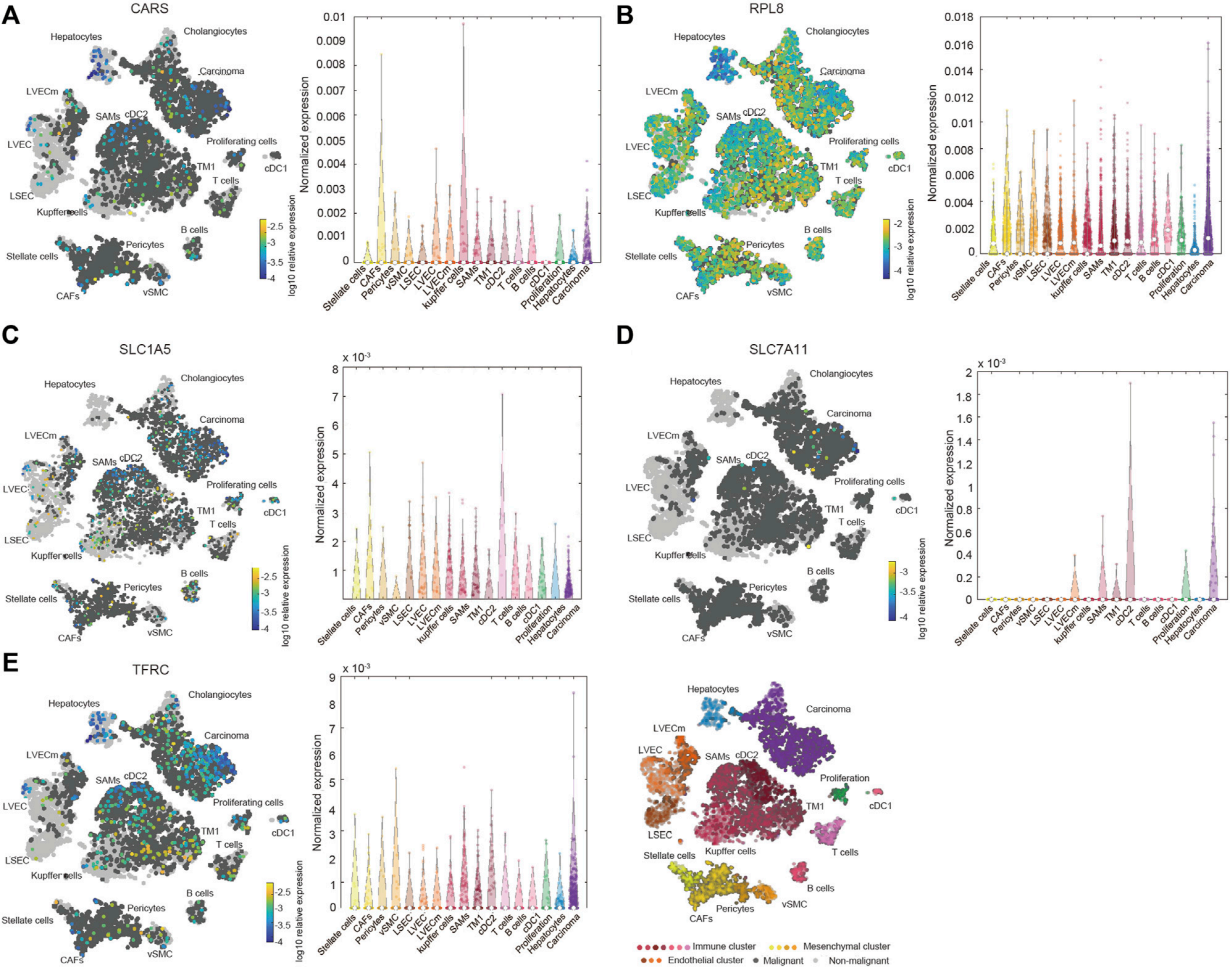
Increased expression and distribution of CARS1 **(A)**, RPL8 **(B)**, SLC1A5 **(C)**, SLC7A11 **(D)** and TFRC **(E)** in HCC according to single-cell RNA-seq analysis using the Human Liver Browse database (https://itzkovitzwebapps.weizmann.ac.il/webapps/home/session.html?app=HumanLiverBrowser). The left tSNE plots show the expression of 5 FRGs in 17 Seurat clusters. The identified clusters are represented by different colors. The light grey dots represent non-malignant cells (normal liver cells), the dark grey dots represent the malignant cells (HCC cells), and the different color dots represent the immune cluster, mesenchymal cluster and endothelial cluster. The right panel shows the normalized expression of each gene in different cells.

**FIGURE 7:**
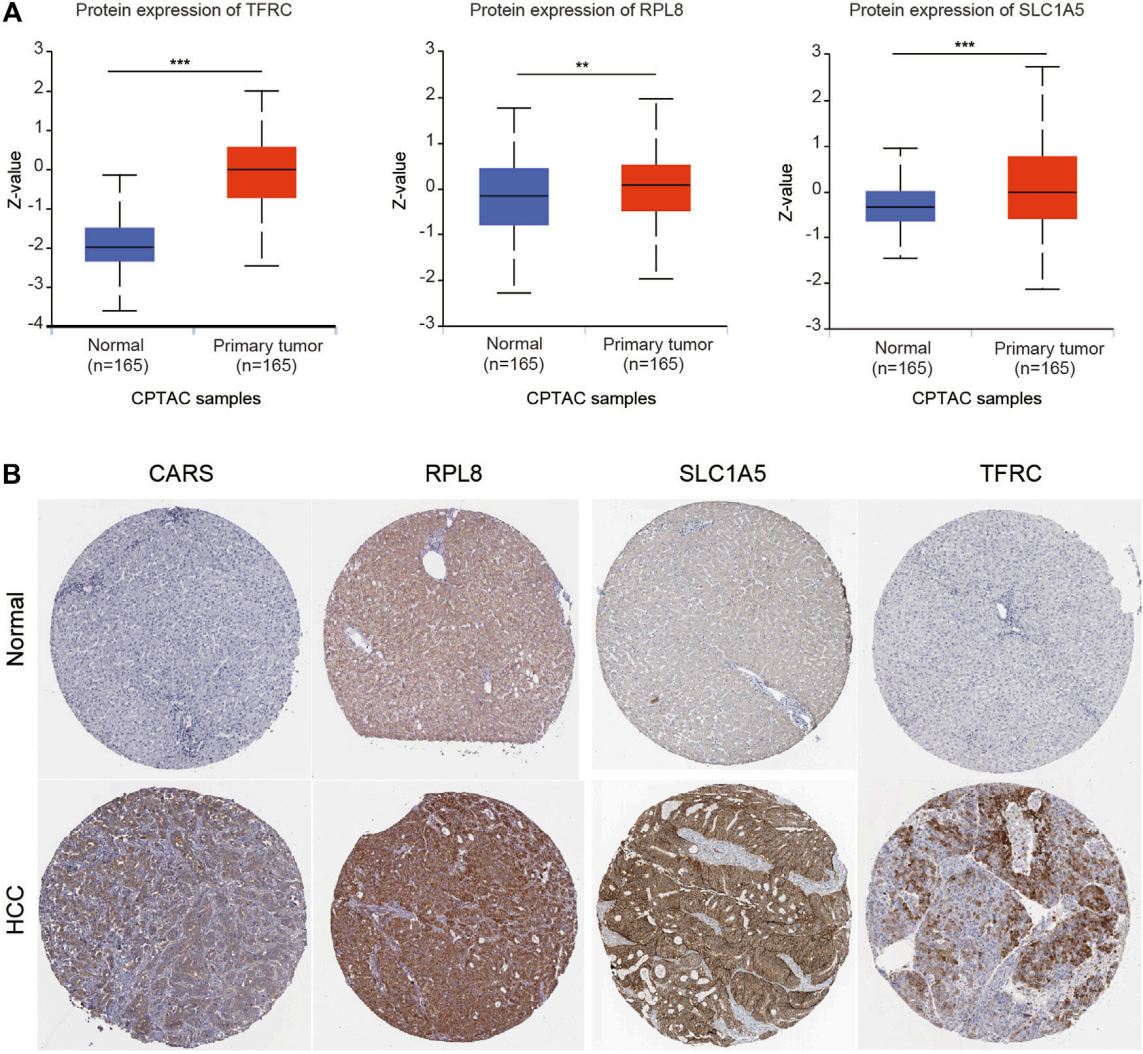
Higher protein expression of FRGs in HCC tissues than in normal liver tissues. **(A)** The protein expression of TFRC, RPL8 and SLC1A5 in HCC tissues and normal tissues was investigated in the CPTAC database. Z-values represent standard deviations from the median across samples for HCC. **(B)** Immunohistochemistry analysis was used to examine the protein expression levels of SLC1A5, TFRC, RPL8 and CARS1 in the HPA database. *p < 0.05, **p < 0.01, ***p < 0.001.

We also investigated the expression of SLC7A11, SLC1A5, TFRC, RPL8 and CARS1 in HCC and normal liver tissues using single-cell RNA-seq data. Higher expression of these five genes was observed in carcinoma cells than in hepatocytes (Figures 6A–E). More importantly, the single-cell RNA-seq analysis results suggested that these genes were not only expressed in liver cells but also expressed in other types of cells, such as cancer-associated fibroblasts (CAFs), T cells and B cells (Figures 6A–E). The protein expression levels of these genes were further evaluated using the Clinical Proteomic Tumor Analysis Consortium (CPTAC) database. The protein levels of TFRC, RPL8 and SLC1A5 were higher in HCC tissues than in normal liver tissues (Figure 7A). The IHC staining results from the HPA database also provided the protein levels of four prognostic FRGs, including TFRC, RPL8, CRAS1 and SLC1A5, between HCC and adjacent normal liver tissues, which were consistent with the CPTAC data (Figure 7B).

### Construction of a Key Ferroptosis-Related Gene Prognostic Signature

**FIGURE 8:**
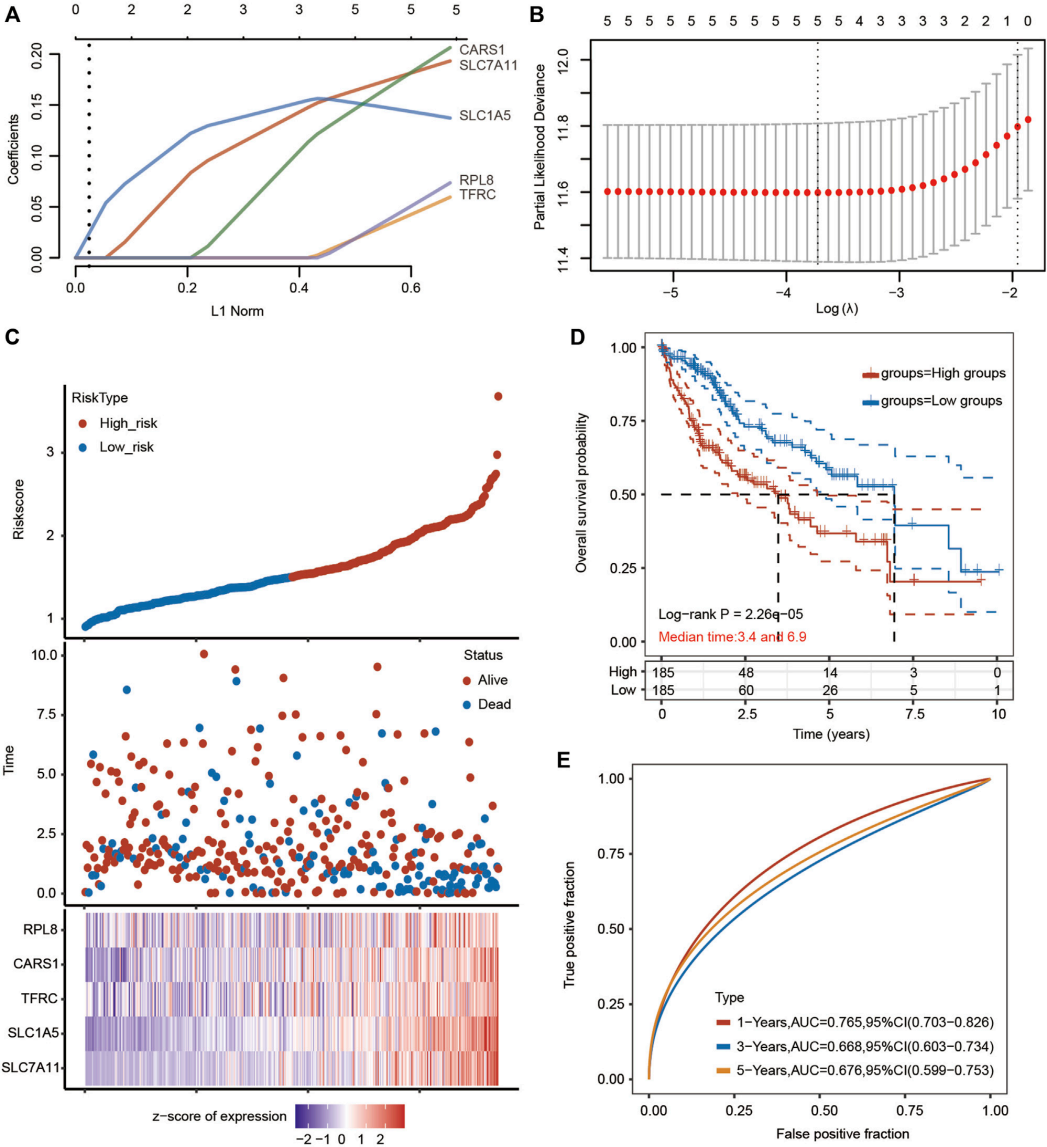
Prognostic analysis of the five-FRG signature model in the TCGA-HCC cohort. **(A,B)** LASSO Cox regression analysis was performed to build a prognostic model based on 5 FRGs. **(C)** The distributions of the risk score and OS status in the TCGA-HCC cohort. **(D)** Kaplan–Meier curves for the OS of HCC patients in the low-risk group and high-risk group in the TCGA-HCC cohort (*p* < 0.001, Log-rank test). **(E)** The AUC of time-dependent ROC curves verified the prognostic performance of the risk score in the TCGA-HCC cohort.

We then performed LASSO Cox regression analysis to construct a gene signature based on the optimum *λ* value (Figures 8A–C). The risk score was calculated as follows: risk score = (0.1668 × SLC7A11) + (0.1507 × SLC1A5) + (0.0221 × TFRC) + (0.1515 × CARS1) + (0.0234 × RPL8). According to the median score calculated by the risk score formula, the HCC patients were divided into two subgroups: the low-risk subgroup and the high-risk subgroup. Importantly, HCC patients in the high-risk subgroup exhibited worse OS than those in the low-risk subgroup (Figure 8D). Receiver operating characteristic (ROC) analysis was used to assess the specificity and sensitivity of the prognostic model. The areas under the ROC curve (AUCs) were 0.764 for 1-year survival, 0.667 for 3-year survival, and 0.674 for 5-year survival (Figure 8E).

### External Validation of the 5-FRG Signature Using the International Cancer Gene Consortium Database

A total of 240 HCC patients from the ICGC database were used for the validation of the prognostic signature. We also established a multivariate LASSO Cox regression model according to the expression information of the five hub FRGs (Supplementary Figures S5A–C). The ferroptosis-related signature was constructed based on the best setting for the tuning parameter *λ*. The formula for the prognostic risk assessment score was as follows: risk score = (0.1343 × SLC7A11 + 0.5613 × TFRC + 0.3277 × RPL8 + 0.2725 × CARS1). The HCC patients in the ICGC dataset were also divided into high-risk and low-risk subgroups. When the risk score increased, the risk of death increased, and the survival time of HCC patients decreased (Supplementary Figure S5C). The patients in the high-risk group had an unfavorable prognosis compared with those in the low-risk group (Supplementary Figure S5). The prognostic efficiency of the five-gene signature was further evaluated by time-dependent ROC curves. The AUC values were 0.766 for 1-year survival, 0.78 for 2- year survival, and 0.771 for 3-year survival (Supplementary Figure S5E).

### Independent Prognostic Value of the 5-FRG Signature Based on Different Clinicopathological Characteristics

**FIGURE 9:**
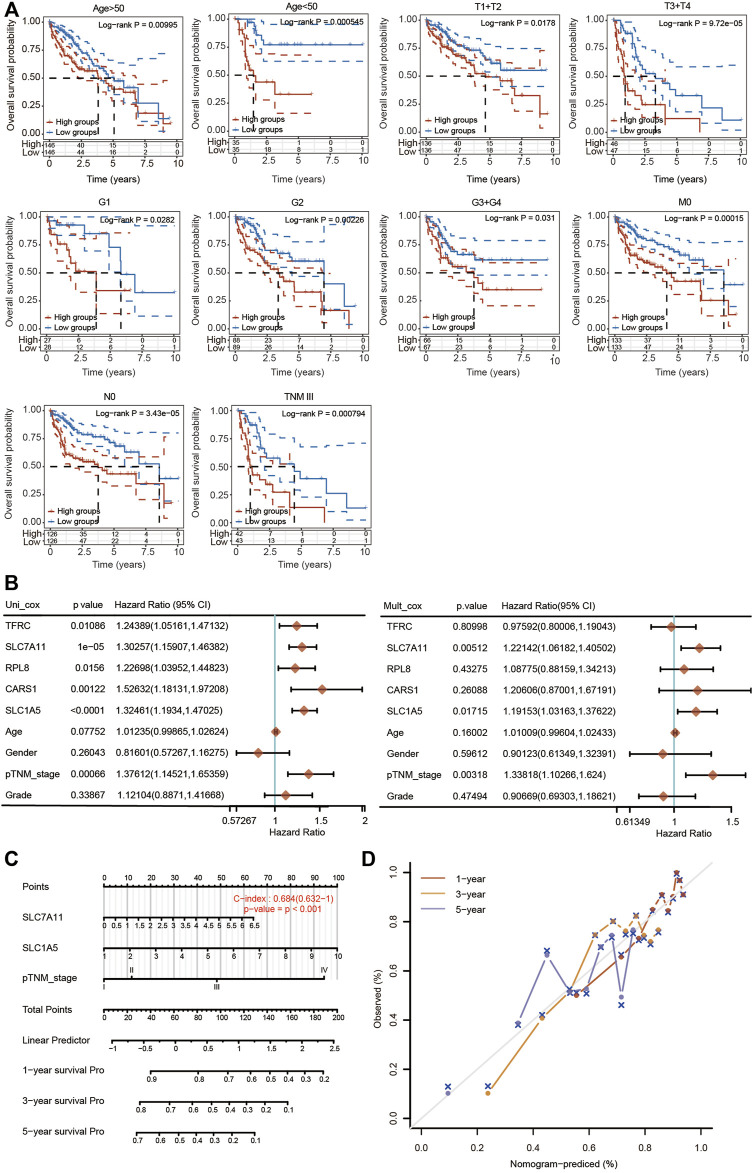
Construction of a nomogram based on five FRGs and clinical factors. **(A)** Kaplan–Meier curves of OS between every two groups stratified by age < 50, age < 50, T1 + T2, T3 + T4, G1, G2, G3 + G4, N0, M0 and TNM stage III (*p* < 0.05, Log-rank test). **(B)** Univariate and multivariate Cox regression analyses of five FRGs and clinical factors are shown. **(C)** A stable and accurate hybrid nomogram that contains clinicopathological characteristics and the novel prognostic signature. Nomogram for predicting 1-, 3-, and 5-year OS. **(D)** Calibration curves were generated and the 1-, 3-, and 5-year predictions of the nomogram were observed to be consistent with the actual survival rates.

The prognostic potential of the 5-FRG signature was further confirmed according to different clinicopathological parameters of HCC. As shown in Figure 9A, the 5-FRG signature was significantly correlated with unfavorable prognosis in young (<50 years) and old (>50 years), early grade (G1 and G2) and advanced grade (G3 + G4), early stage (T1 + T2) and advanced stage (T3 + T4), M0, N0, and TNM stage III patients with HCC. These results imply that the 5-FRG signature according to the risk grouping can serve as a useful tool for predicting the survival of HCC patients among each stratum of age, grade and stage.

We then performed univariate and multivariate Cox regression analyses to further assess the prognostic value of the 5 FRGs in HCC patients (Figure 9B). According to the results of univariate Cox regression analysis, TFRC, SLC7A11, RPL8, CARS1, SLC1A5 and TNM stage were obviously correlated with the OS of HCC patients. According to the results of multivariate Cox regression analysis, SLC7A11, SLC1A5 and TNM stage showed significant correlations with the OS of HCC patients (Figure 9B).

Based on the multivariate regression analysis, we constructed a novel nomogram model integrating SLC7A11, SLC1A5 and TNM stage to predict the 1-, 3- and 5-year OS rates of patients with HCC (Figure 9C). The C-index of the prognostic nomogram was 0.684 (Figure 9C). The calibration plots of the nomogram showed good agreement between the actual and nomogram-predicted 1-, 3- and 5-year survival rates (Figure 9D).

### Associations of the Risk Score With Immune Cell Infiltration in Hepatocellular Carcinoma

**FIGURE 10:**
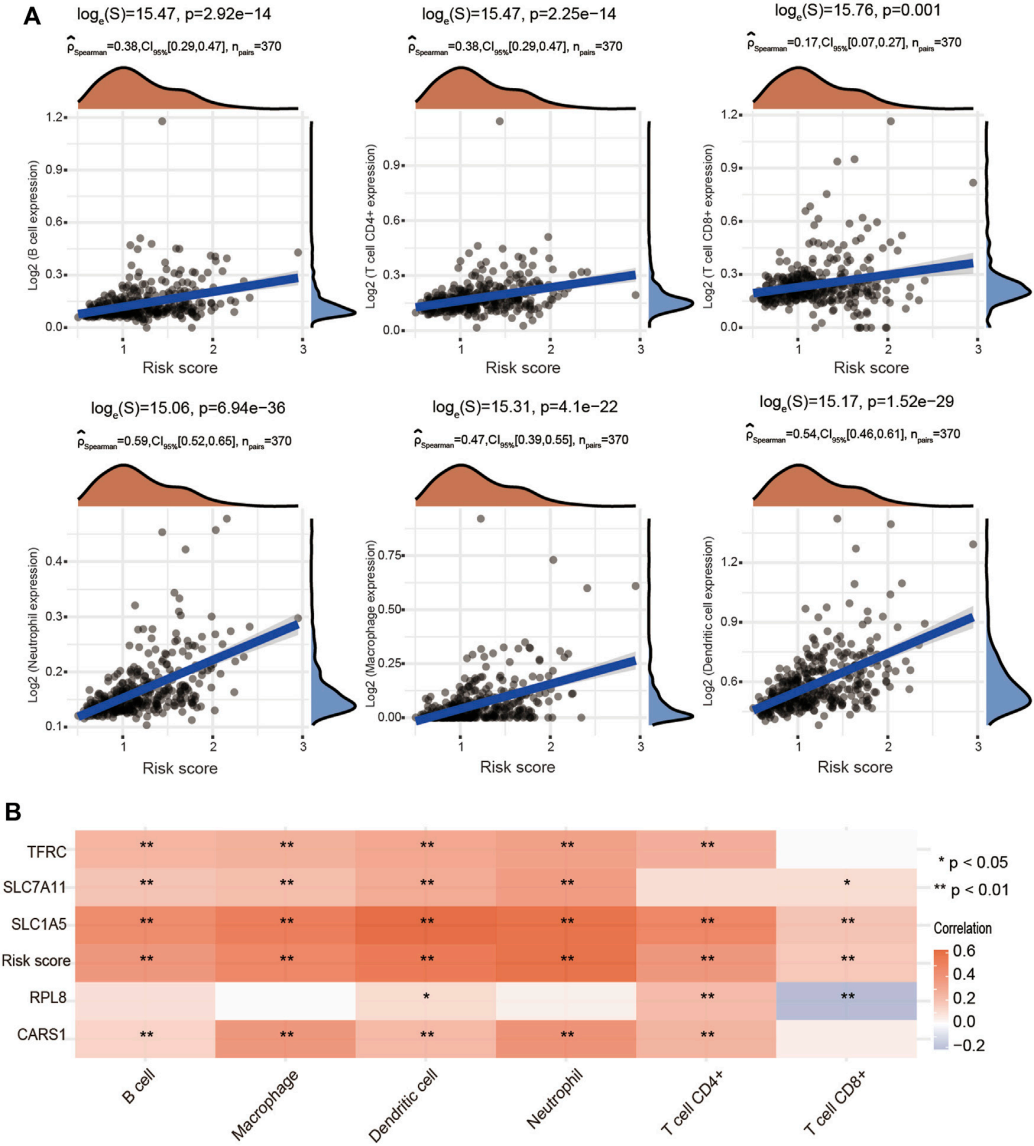
Positive association of the risk score with immune cell infiltration in HCC. **(A)** Correlations between the risk score and the infiltration levels of B cells, CD4+ T cells, CD8+ T cells, macrophages, neutrophils and dendritic cells using the spearman analysis. **(B)** Heatmap showing the correlation between risk score and five ferroptosis-related genes and the infiltration levels of six major immune cells. Red represents a positive correlation and blue represents a negative correlation. *p < 0.05, **p < 0.01.

To further explore whether the risk score is correlated with the characteristics of the TME, we estimated the relationship between the infiltration level of different immune cells and the risk score using the TIMER algorithm. We observed significant and positive correlations between the ferroptosis-related risk score and the infiltration levels of B cells, CD4+ T cells, CD8+ T cells, neutrophils, macrophages and dendritic cells (Figure 10A). We also found that SLC7A11, SLC1A5, CARS1 and TFRC were obviously associated with the infiltration of most types of immune cells (Figure 10B).

### Genetic Mutation and Drug Sensitivity of the 5 Ferroptosis-Related Genes in Hepatocellular Carcinoma

**FIGURE 11:**
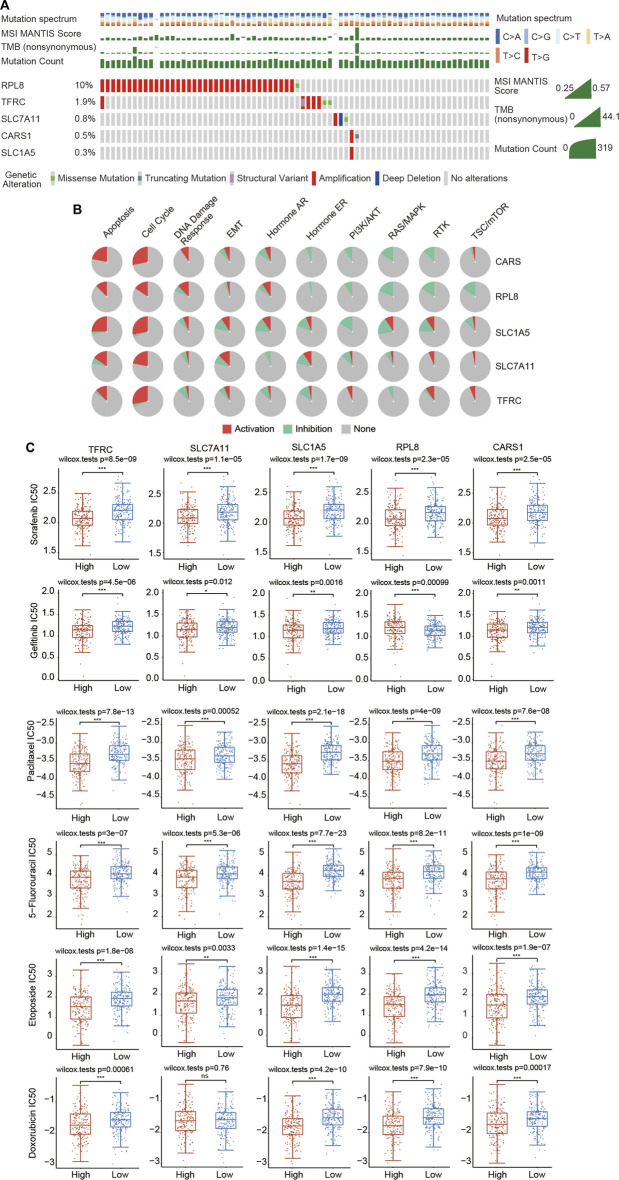
Landscape of the genetic alterations of the 5 FRGs and upregulated expression of the 5 FRGs is correlated with drug sensitivity in HCC. **(A)** The genetic alteration rates of CARS1, RPL8, TFRC, SLC1A5 and SLC7A11 in HCC were examined in the cBioPortal database. **(B)** The correlations between 5 FRGs and various cancer-related pathways were examined in the GSCALite database. **(C)** The correlations between the expression of 5 FRGs and drug sensitivity was investigated according to the GDSC database using the R package pRRophetic. Higher expression of the 5 FRGs was significantly correlated with lower IC50 values of these well-known chemotherapy drugs. The difference was compared statistically with the Wilcoxon test. *p < 0.05, **p < 0.01, ***p < 0.001.

Genome-wide analysis of the genetic mutations of these five genes was explored through the cBioPortal database. The RPL8 gene exhibited the highest mutation frequency (10%), followed by TFRC (1.9%), SLC7A11 (0.8%), CARS1 (0.5%) and SLC1A5 (0.3%) (Figure 11A; Supplementary Figure S6A). The most common type of genetic alteration was amplification. We then analyzed the association between these five FRGs and common oncogenesis-related pathways, including the apoptosis, cell cycle, DNA damage response, EMT, hormone AR, hormone ER, PI3K/AKT, RAS/MAPK, RTK and TSC/mTOR pathways. We found that the hub FRGs were significantly correlated with the activation or inhibition of these pathways (Figure 11B; Supplementary Figure S6B).

Chemotherapy is still commonly used in the comprehensive treatment for HCC. We then estimated whether these 5 FRGs could affect the sensitivity of chemotherapeutic agents commonly used for treating HCC, including sorafenib, gefitinib, paclitaxel, 5-fluorouracil, etoposide, doxorubicin, vinblastine, docetaxel, gemcitabine, cytarabine, AKT inhibitor VIII, sunitinib and metrhotrexate according to the GDSC database using the R package pRRophetic. Based on the median expression level of the 5 FRGs, all of the HCC patients were divided into the high-expression group and low-expression group. We found that a significant difference in the IC50 existed between the high- and low-expression groups for all these chemotherapeutic drugs (Figure 11C; Supplementary Figure S7). HCC patients with high expression of 5 FRGs were more sensitive to these well-known chemotherapy drugs (Figure 11C; Supplementary Figure S7). These data suggest that these five genes affect the actual therapeutic effect of these drugs in HCC through ferroptosis.

Immunotherapy targeting immune checkpoints has emerged as a novel and rapidly developing therapeutic approach for the clinical treatment of multiple tumor types. The high expression of the 5 FRGs was associated with high expression of most immune checkpoints, such as CTLA-4, PD-1 and PD-L1 (Supplementary Figure S8A). According to the TIDE algorithm, we found that upregulated expression of SLC1A5 and RPL8 corresponded to a high TIDE score, indicating that decreasing FRG expression may improve the therapeutic effect of immunotherapy (Supplementary Figure S8B). Taken together, these results indicate that these 5 hub genes could serve as potential biomarkers for drug screening and provide additional targets for the immunotherapy of HCC.

## Discussion

Liver cancer is a commonly diagnosed malignancy worldwide and has a high mortality rate among all human tumors (Forner et al., 2018; Sung et al., 2021). Tremendous efforts have been made to develop novel strategies against liver cancer. However, the OS of HCC patients is still unsatisfactory (Sung et al., 2021). Resistance to chemotherapy has been widely recognized as an important cause of death in cancer patients. The in-depth and systematic elucidation of the underlying molecular mechanisms of liver cancer could open new horizons for its treatment. Ferroptosis, a newly identified type of programmed cell death, occurs in an iron-dependent manner, peroxidizes unsaturated phospholipids and causes the accumulation of lethal ROS (Stockwell et al., 2017). The subsequent oxidative damage and oxidative stress imbalance induced by ferroptosis contribute to cell death in multiple cancer cells (Bebber et al., 2020; Chen X. et al., 2021). In fact, initiating ferroptosis pathways may be able to overcome existing chemotherapeutic resistance and open a new therapeutic area for cancer treatment (Zhao et al., 2022). Therefore, understanding the molecular mechanism of ferroptosis in HCC is a promising therapeutic strategy for liver cancer that selectively induces ferroptosis. Here, using data from the TCGA, ICGC and GEO datasets, we performed extensive bioinformatics analyses to build a risk model composed of five FRGs, which were significantly correlated with the prognosis of patients with HCC. The predictive significance of the constructed signature was further investigated by exploring the ferroptosis-related oncogenic roles and immunological response in HCC patients. This ferroptosis risk model may provide clinicians with more intuitive and rational information for the treatment of HCC in the future.

In the present study, three independent subtypes were comprehensively identified by consensus clustering analysis based on the expression level of 25 FRGs in HCC. There were significant differences in terms of T stage, TNM stage and grade among these three clusters (Figure 3). Moreover, cluster 3 exhibited higher expression and unfavorable prognosis compared with clusters 1 and 2 (Figure 3). Next, prognosis-related ferroptosis regulators were selected and used to construct a novel risk signature to predict the OS of HCC patients. A risk model of 5 FRGs (SLC7A11, SLC1A5, CARS1, RPL8 and TFRC) was developed and validated on the basis of the differential FRGs among these three clusters (Figure 5). Patients with HCC were further divided into high-risk and low-risk groups according to the median value of the risk model. Multivariate Cox regression analysis indicated that the FRG risk model was an independent prognostic model of OS (Figure 9). ROC analysis further suggested the superiority of our risk model in predicting the OS of HCC patients compared with established clinicopathological characteristics. We then extended our risk model and built a nomogram to predict 1-year, 3-year and 5-year OS. The calibration curve exhibited excellent agreement between the model predictions and the actual OS (Figure 9). Therefore, our risk model, based on five FRGs, has a high degree of accuracy and may contribute to the search for new biomarkers of HCC.

The development of new drugs for cancer is a long and tortuous process, inevitably involving thousands of failures. Drugs that are currently available have already passed safety assessments, which means that research on these drugs to treat other cancers can save time and money. Sorafenib is a novel oral kinase inhibitor that targets various tyrosine kinases. Based on favorable data from preclinical and clinical trials, sorafenib has been approved as a first-line treatment for advanced HCC. However, it is still necessary to further understand the role of sorafenib in the treatment of HCC and to explore more effective drug combination regimens. Based on evidence that the cytotoxic effect of sorafenib on liver cancer cells Huh7 was significantly inhibited by the iron chelator deferoxamine and the ferroptosis-specific inhibitor ferrostatin-1, sorafenib was proposed as a ferroptosis-inducing agent in 2013 (Louandre et al., 2013). Subsequently, it was found that sorafenib triggers ferroptosis by inhibiting the System Xc-, while beta-mercaptoethanol (β-ME), deferoxamine, and ferrostatin-1 can restore sorafenib-induced cell death (Louandre et al., 2013; Sun et al., 2016b). In addition, sorafenib induces ferroptosis in renal adenocarcinoma ACHN and liver cancer HepG2 cells (Lachaier et al., 2014; Houessinon et al., 2016; Sun et al., 2016a). In addition to sorafenib, various chemotherapeutic drugs commonly used for treating liver cancer, including gefitinib, paclitaxel, 5-fluorouracil, etoposide, doxorubicin, vinblastine, docetaxel, gemcitabine, cytarabine, AKT inhibitor VIII, sunitinib and metrhotrexate, by blocking the cell cycle, promoting cell death, suppressing specific signaling pathways and inhibiting angiopoiesis. Here, we showed that 5 FRGs were significantly associated with the sensitivity of these drugs according to the GDSC database (Figure 11). The GDSC database records approximately 1,000 different human cancer cell lines and screens them with more than 100 existing anticancer drugs. We found that in the high FRG expression group, the IC50 values of various chemotherapeutics and small-molecule anticancer drugs were significantly lower, demonstrating that HCC patients with high expression of 5 FRGs might obtain more therapeutic benefit from these drugs (Figure 11C; Supplementary Figure S7). These data suggest that these five genes may affect the actual therapeutic effect of these drugs in HCC through ferroptosis. In future research, the development of drugs targeting these five genes may result in a more effective treatment for liver cancer with fewer side effects.

Previous studies have suggested that ferroptosis is immunogenic and induces an inflammatory immune response (Chen X. et al., 2021; Zhao et al., 2022). Cells undergoing ferroptosis can often interact with the immune system in multiple ways through complex cellular and molecular mechanisms. For example, ferroptotic cells can release damage-associated molecular patterns (DAMPs) or lipids that act as messengers for surrounding immune cells (Friedmann Angeli et al., 2019; Chen X. et al., 2021). The signals released can be immunogenic or inflammatory, leading to the activation of various immune responses. Furthermore, danger signals in the TME may affect the function of immune cells and contribute to tumor immune escape. Interestingly, the interaction between ferroptosis and anticancer immunotherapy is multifaceted. A recent study revealed the role of ferroptosis in CD8+ T cell-mediated antitumor immunity. Immune checkpoint inhibitor treatment activates CD8+ T cells to produce interferon gamma (IFNγ) and promotes ferroptosis in tumor cells (Wang et al., 2019). The underlying mechanism of this effect is that IFNγ is recognized by receptors on the tumor cell membrane, increasing the expression of the transcription factor STAT1, which in turn downregulates the expression of SLC7A11 and SLC3A2, resulting in the inhibition of the GSH/GPX4 axis, and finally sensitizing tumor cells to ferroptosis (Wang et al., 2019). In addition, IFNγ secreted by CD8+ T cells, which are activated by immunotherapy binds to IFNR, activates the JAK-STAT1-IRF1 signaling axis, and induces the expression of ACSL4 in tumor cells (Liao et al., 2022). Increased ACSL4 expression synergizes with arachidonic acid (AA) treatment to trigger potent ferroptosis in various cancer cell lines in an ACSL4-dependent manner (Liao et al., 2022). Thus, ferroptosis has been clearly proven to play an important role in antitumor immunity, not only participating in the elimination of tumor cells by T cells activated by immune checkpoint inhibitors, but also directly affecting the functions of various immune cells, suggesting that ferroptosis may synergize with immunotherapy to inhibit tumor development and progression. Undoubtedly, a better understanding of the interplay between ferroptosis and immunity will greatly facilitate the development of new therapeutic strategies with translational and applied potential.

Chemotherapy and immunotherapy are crucial adjunctive therapies for HCC. PD-1/PD-L1 checkpoint inhibitors have emerged as a promising therapeutic strategy for HCC. In September 2017, the anti-PD-1 antibody nivolumab (Opdivo) was approved by the FDA for the second-line treatment of sorafenib-pretreated patients with advanced liver cancer (El-Khoueiry et al., 2017). Separately, in a phase 2 study, the anti-PD-1 inhibitor pembrolizumab (Keytruda) was investigated as second-line therapy in patients with advanced HCC following sorafenib failure (Zhu et al., 2018). The results confirmed an objective response rate of 17% in HCC patients; thus, the FDA approved pembrolizumab in November 2018 for the treatment of HCC patients who had previously received sorafenib (Zhu et al., 2018; Macek Jilkova et al., 2019). However, the harsh reality shows that only a minority of patients with liver cancer can benefit from this treatment. To enhance the efficacy of immune checkpoint inhibition in HCC patients, future strategies may require predictive factor-based selection to identify which patients may be more sensitive to immunotherapy and combination strategies to improve the antitumor efficacy and clinical success. However, very little has been described about predictive biomarkers of the response to PD-1/PD-L1 blockade in HCC (Macek Jilkova et al., 2019). To date, there are few studies on predictive biomarkers of immunotherapy response in HCC. A previous study demonstrated that deletion of SLC1A5 affects T-cell effector function, resulting in the impaired differentiation of helper T cells into Th1 and Th17 subsets (Nakaya et al., 2014). C-myc is essential for NK-cell metabolism and T-cell activation (Wang et al., 2011; Loftus et al., 2018). Glutamine is extremely important for the immune system, maturation and the differentiation of immune cells. Glutamine uptake through SLC1A5 is necessary for c-myc induction in cytokine-stimulated NK cells (Wang et al., 2011; Loftus et al., 2018). Moreover, recent studies demonstrated that SLC1A5 expression was positively and significantly associated with the abundance of tumor-infiltrating immune cells in stomach adenocarcinoma (STAD) and HCC (Zhao et al., 2021; Zhu et al., 2022). SLC1A5 expression was positively correlated with the expression of PD-1, but negatively correlated with the expression of PD-L1. Therefore, SLC1A5 may have an important role in the TME by modulating immune cell infiltration in STAD and HCC (Zhao et al., 2021; Zhu et al., 2022). In addition, a previous a study suggested that enhanced expression of CARS1 was strongly correlated with unfavorable prognosis in various cancers and was associated with the expression of immune checkpoint genes, including PD-L1 (Wang S. et al., 2021). Thus, CARS1 may be a potential novel prognostic biomarker and novel target for cancer immunotherapy. A recent study demonstrated that patients and a mouse model with a homozygous mutation in *TFRC* showed combined immunodeficiency characterized by decreased proliferation of T and B cells and defective class switching (Jabara et al., 2016). TFRC is also associated with the formation of immunological synapses in T cells during TCR engagement. Our previous study also indicated that TFRC expression was closely and positively correlated with the infiltration levels of many immune cells in breast cancer (Chen F. et al., 2021). We observed that the infiltrating levels of CD8+ T cells, CD4+ T cells, neutrophils, macrophages and dendritic cells were higher in the TFRC high-expression group than in the TFRC low-expression group (Chen F. et al., 2021). Moreover, the expression of LAG3, CTLA-4, PD-L1, CD274, TIGIT, HAVCR2 and PDCD1LG2 was upregulated in the TFRC high-expression group compared with the TFRC low-expression group in breast cancer (Chen F. et al., 2021).

In the present study, CIBERSORT analysis revealed that the three clusters had different infiltrating levels of naïve B cells, memory B cells, CD8+ T cells, Tregs, T follicular helper cells, resting NK cells, monocytes, M0 macrophages, M1 macrophages, activated mast cells, resting mast cells and neutrophils. Interestingly, we subsequently identified that cluster 3 showed higher expression of immune checkpoints, including CD274, CTLA4, HAVCR2, LAG3, PDCD1, PDCD1LG2 and TIGIT (Figure 4). More importantly, in the present study, the single-cell sequencing analysis results implied that SLC7A11, SLC1A5, CARS1, RPL8 and TFRC were not only upregulated in liver cancer cells but also expressed in immune cells. Moreover, the expression of the 5 FRGs was positively correlated with the expression of most immune checkpoints, including CTLA-4, PD-1 and PD-L1 (Supplementary Figure S8A). We also observed that increased expression of SLC1A5 and RPL8 corresponded to a high TIDE score based on the TIDE algorithm, suggesting that decreasing FRG expression may improve the therapeutic effect of immunotherapy (Supplementary Figure S8B). Taken together, these findings identify the essential roles of these 5 genes in regulating the immune response and immune cell infiltration in cancer.

Inevitably, there are several limitations in the present study. First, we constructed a prognostic model based on differential FRGs to predict the survival rate of patients with HCC according to retrospective data from the TCGA database. Although we validated our risk model using the ICGC cohort, more large-scale data to verify the clinical application value of our FRG-based survival model are needed. Second, although the expression levels of 5 FRGs in HCC and normal tissues were investigated using TCGA, ICGC and GEO datasets, the expression levels and scores of clinical samples are needed. Third, although the correlations between the 5 FRGs and immune cell infiltration were observed from our analyses, the correlation coefficient was not strong. Thus, the potential relationships between the prognostic signature and immune infiltration need to be experimentally validated. Forth, when the consensus clustering analysis was performed, although we identified that *k* = 3 as the optimal clustering stability, there was some crossover of the samples in the three clusters. Clearer cluster separation and a more satisfactory classification of HCC samples will make the conclusion more convincing.

## Conclusion

In the present study, we comprehensively analyzed the clustering effects of ferroptosis in patients with HCC based on the expression levels of reported FRGs. Three clusters of patients with HCC suggested a prominent prognostic difference, and a risk model of 5 FRGs was developed on the basis of the differential FRGs among the three clusters. Although similar risk models of FRGs have been established in HCC patients, our analyses provided a new method for discovering new prognosis-associated genes in HCC by differential analysis in three clusters. Our analysis results indicated that the 5 FRGs of the risk model were significantly associated with the OS of HCC patients.

## Data Availability

The datasets presented in this study can be found in online repositories. The names of the repository/repositories and accession number(s) can be found in the article/Supplementary Material.

## Supplementary Material

Supplementary Figure S1 Confirmation of the upregulated or downregulated expression of FRGs based on the ICGC database. **(A)** Heatmap showing the differential expression of 25 FRGs in normal individuals and HCC patients. Blue represents tumor tissues and red represents the normal tissues. The difference in gene expression was compared statistically with the Wilcoxon test. **(B)** Violin plots showing the differential expression of 25 FRGs in normal individuals and HCC patients. Red represents tumor tissues and blue represents the normal tissues. The interquartile range of values is represented by the upper and lower ends in the box and the median value is represented by the lines in the boxes. The difference in gene expression was compared statistically with the Wilcoxon test. **(C)** Correlation and prognostic values of FRGs in HCC. The interactions between genes were shown by linking lines and the correlation strength between genes was shown by thickness. The effect of each FRG on the prognosis was indicated by the size of the circle and Log-rank test. The red and blue lines represent positive and negative correlations, respectively.

Supplementary Figure 2 Increased or decreased expression of FRGs in HCC patients based on GEO datasets. **(A)** Violin plots showing the differential expression of FRGs in normal individuals and HCC patients in the GSE10143 dataset. **(B)** Violin plots showing the differential expression of FRGs in normal individuals and HCC patients in the GSE36376 dataset.

Supplementary Figure 3
**(A)** The differential expression of 24 FRGs in three clusters. The difference in gene expression is compared statistically with the Kruskal–Wallis test. The interquartile range of values is represented by the upper and lower ends in the box and the median value is represented by the lines in the boxes. The outliers are shown as dots. **(B)** Potential immunotherapy response in three clusters was predicted with the Tumor Immune Dysfunction and Exclusion (TIDE) algorithm using the R packages ggplot2 and ggpubr. The difference in TIDE scores was compared statistically with the Kruskal–Wallis test. **p* < 0.05, ***p* < 0.01, ****p* < 0.001.

Supplementary Figure 4 Differential expression of CARS1, RPL8, TFRC, SLC1A5 and SLC7A11 in GSE10143. The difference in gene expression was compared statistically with the Wilcoxon test. ****p* < 0.001.

Supplementary Figure 5 Confirmation of the prognostic value of the 5 FRG signature model in the ICGC cohort. **(A,B)** LASSO Cox regression analysis was performed to build a prognostic model based on the 5 FRGs. **(C)** The distributions of the risk score and OS status in the TCGA dataset. **(D)** Kaplan–Meier curves for the OS of HCC patients in the low-risk group and high-risk group in the ICGC cohort (*p* < 0.001, Log-rank test). **(E)** The AUCs of time-dependent ROC curves verified the prognostic performance of the risk score in the ICGC dataset.

Supplementary Figure 6 Correlations between the 5 FRGs and genetic alterations and cancer pathways. **(A)** The genetic alteration rates of CARS1, RPL8, TFRC, SLC1A5 and SLC7A11 were examined in the cBioPortal database. **(B)** Pathway analysis of 5 FRGs was performed in the GSCALite database. Network diagram indicating the correlations between FRGs and multiple cancer pathways.

Supplementary Figure 7 Chemosensitivity difference provides potential therapeutic targets. The correlations between the expression of 5 FRGs and drug sensitivity was investigated according to the GDSC database using the R package pRRophetic. Higher expression of the 5 FRGs was significantly correlated with lower IC50 values of these well-known chemotherapy drugs. The difference was compared statistically with the Wilcoxon test. **p* < 0.05, ***p* < 0.01, ****p* < 0.001.

Supplementary Figure 8 The 5 FRGs affect immune checkpoint gene expression and immunotherapy response in HCC. **(A)** The different expression levels of immune checkpoint genes in the high- or low-expression groups. The difference in gene expression was compared statistically with the Wilcoxon test. **(B)** Potential immunotherapy response in the high- or low-expression groups was predicted with the TIDE algorithm using the R packages ggplot2 and ggpubr. Upregulated expression of SLC1A5 and RPL8, but not TFRC, SLC7A11 and CARS1, corresponded to a high TIDE score. The difference of the TIDE scores was compared statistically with the Wilcoxon test. **p* < 0.05, ***p* < 0.01, ****p* < 0.001.
