# Supplementary figures and images for: Development and Validation of a Novel Ferroptosis-Related Gene Signature for Prognosis and Immunotherapy in Hepatocellular Carcinoma
Source: Front Mol Biosci. 2022 Jun 30;9:940575. doi: 10.3389/fmolb.2022.940575 (PMC9280137; doi:10.3389/fmolb.2022.940575)

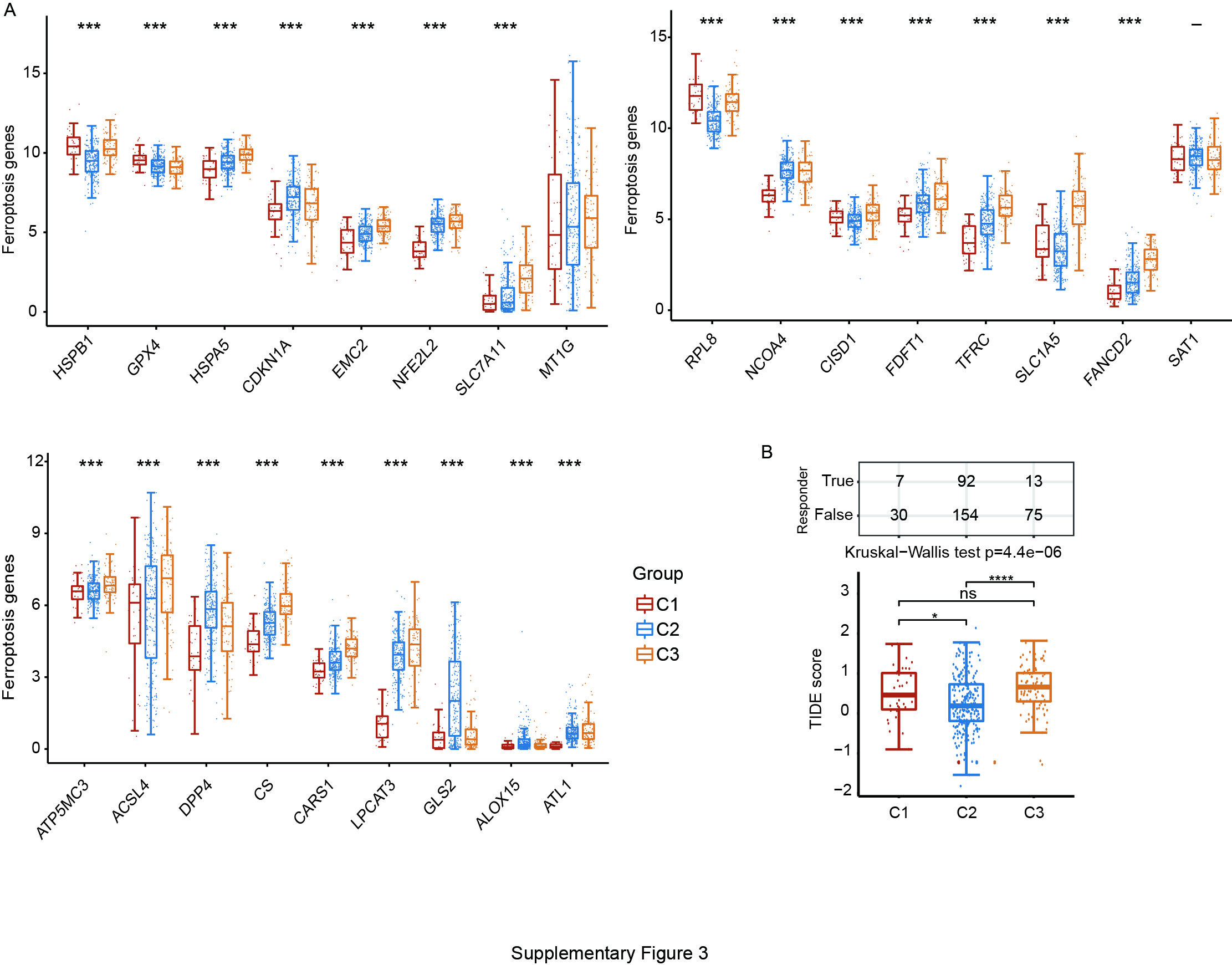

Supplement: Supplementary file 1 [file Image3.JPEG]

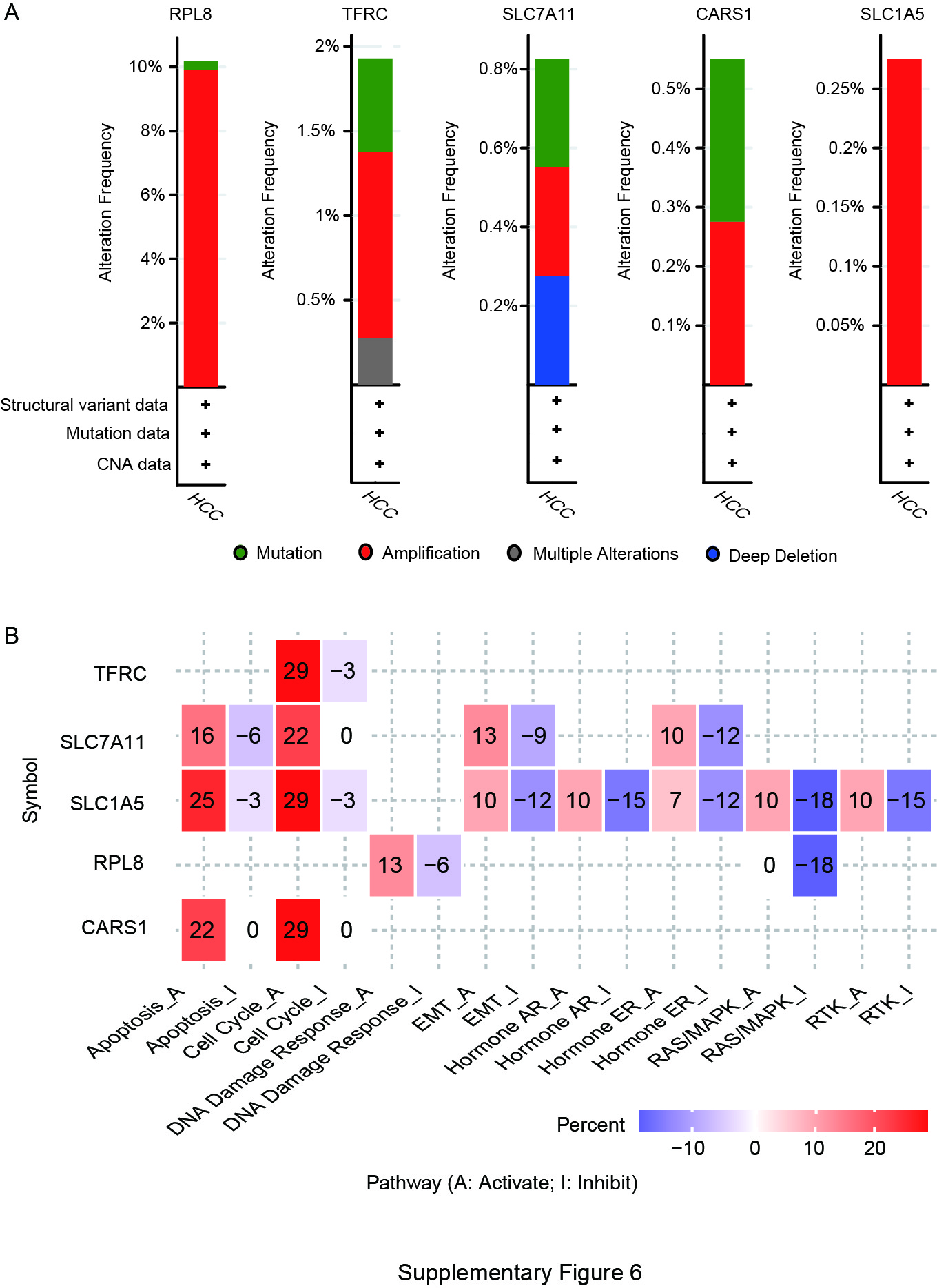

Supplement: Supplementary file 2 [file Image6.jpg]

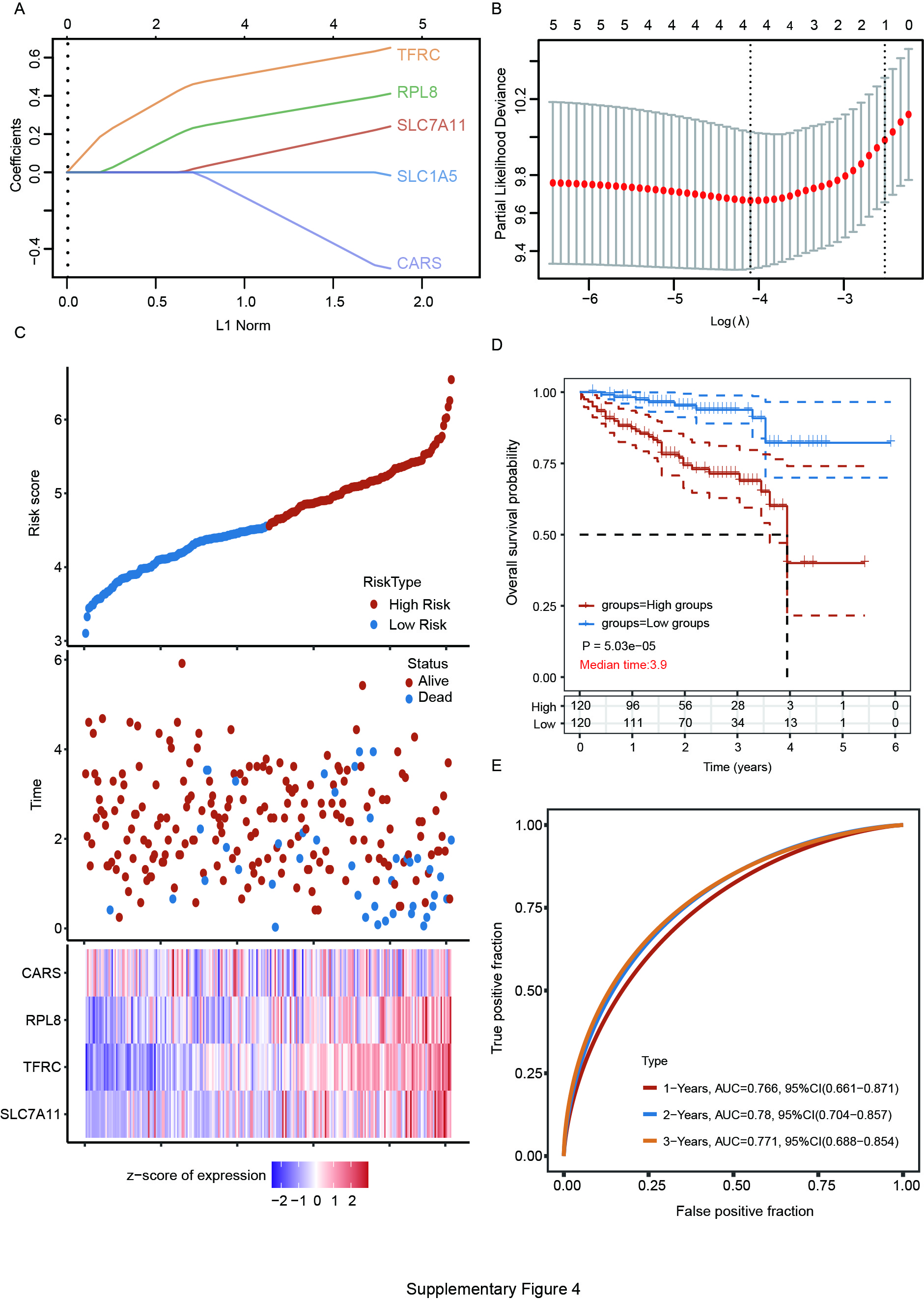

Supplement: Supplementary file 3 [file Image4.JPEG]

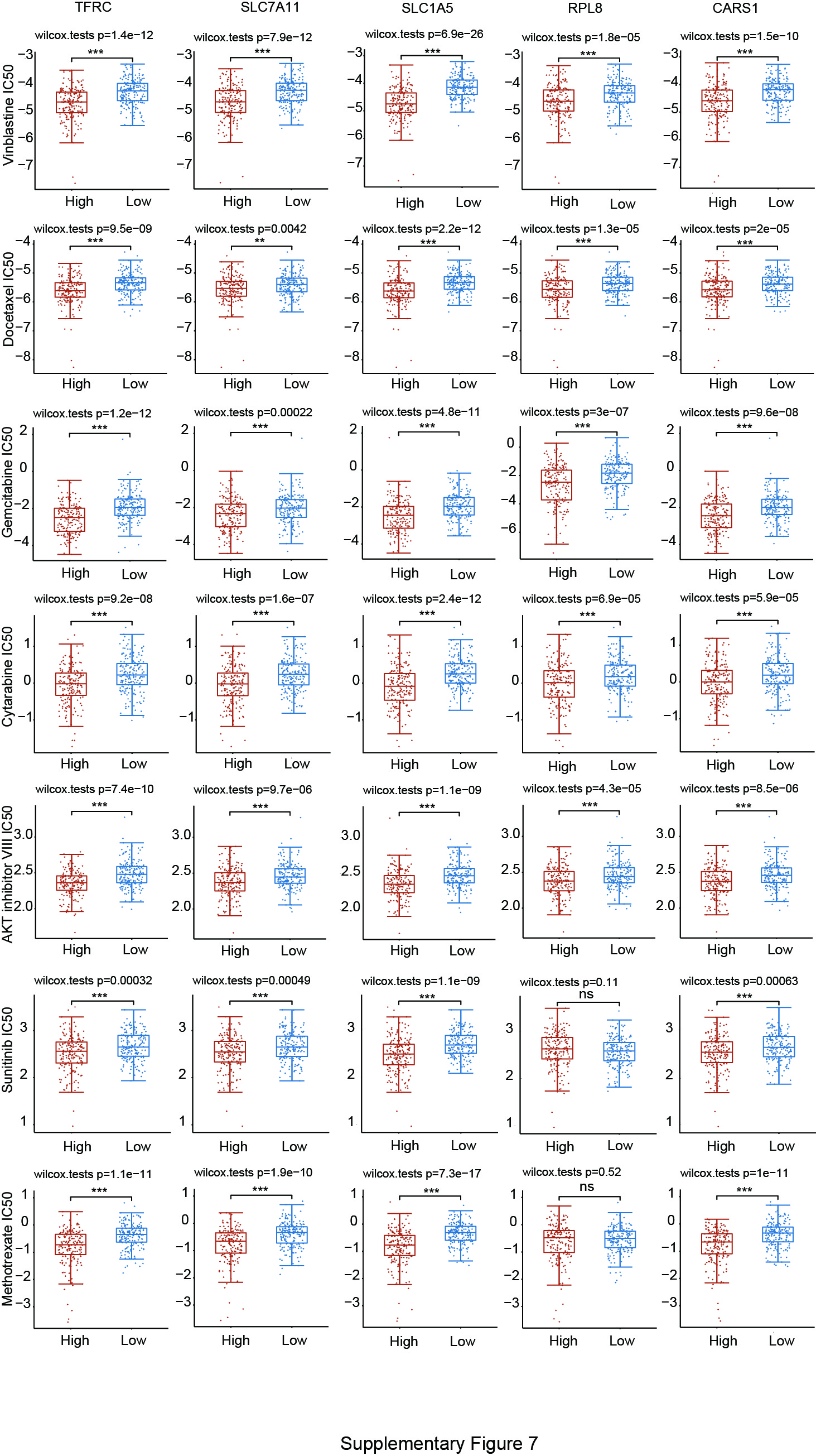

Supplement: Supplementary file 4 [file Image7.JPEG]

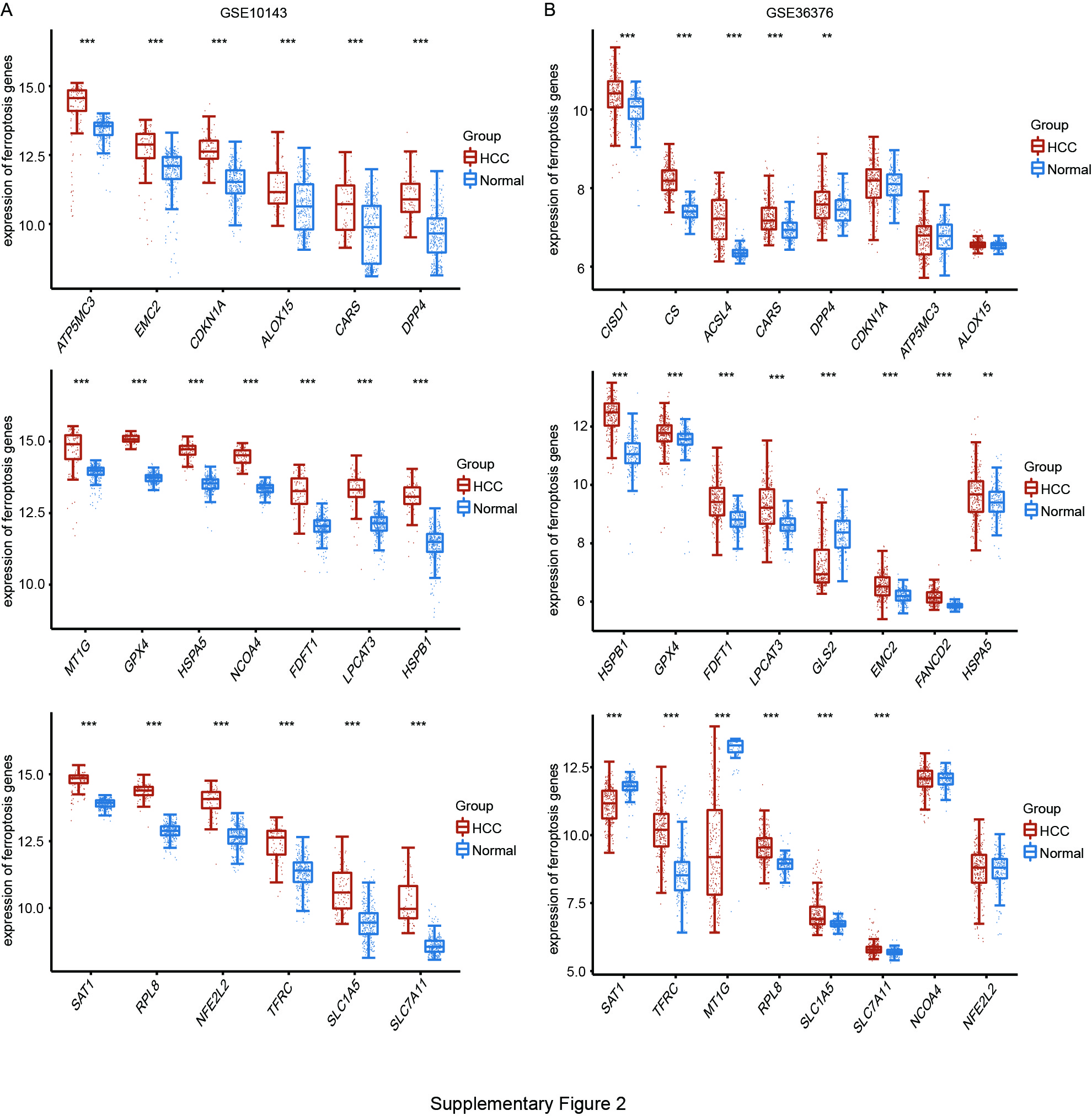

Supplement: Supplementary file 5 [file Image2.JPEG]

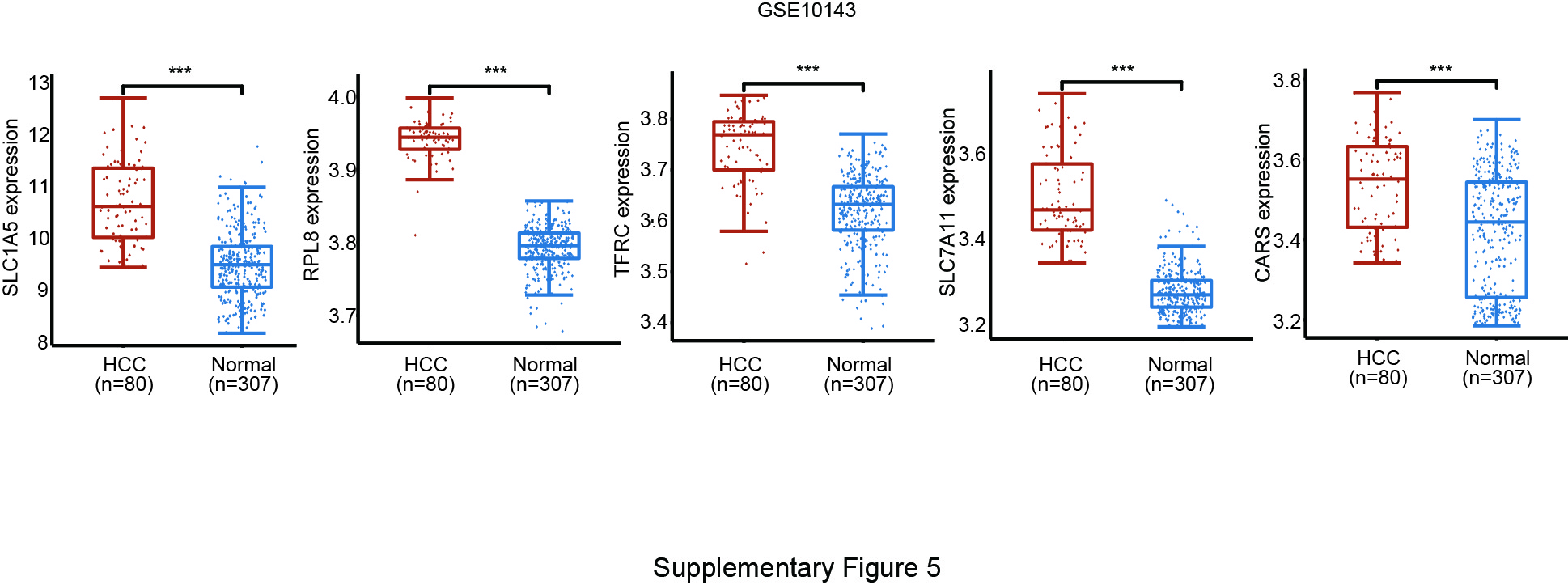

Supplement: Supplementary file 6 [file Image5.JPEG]

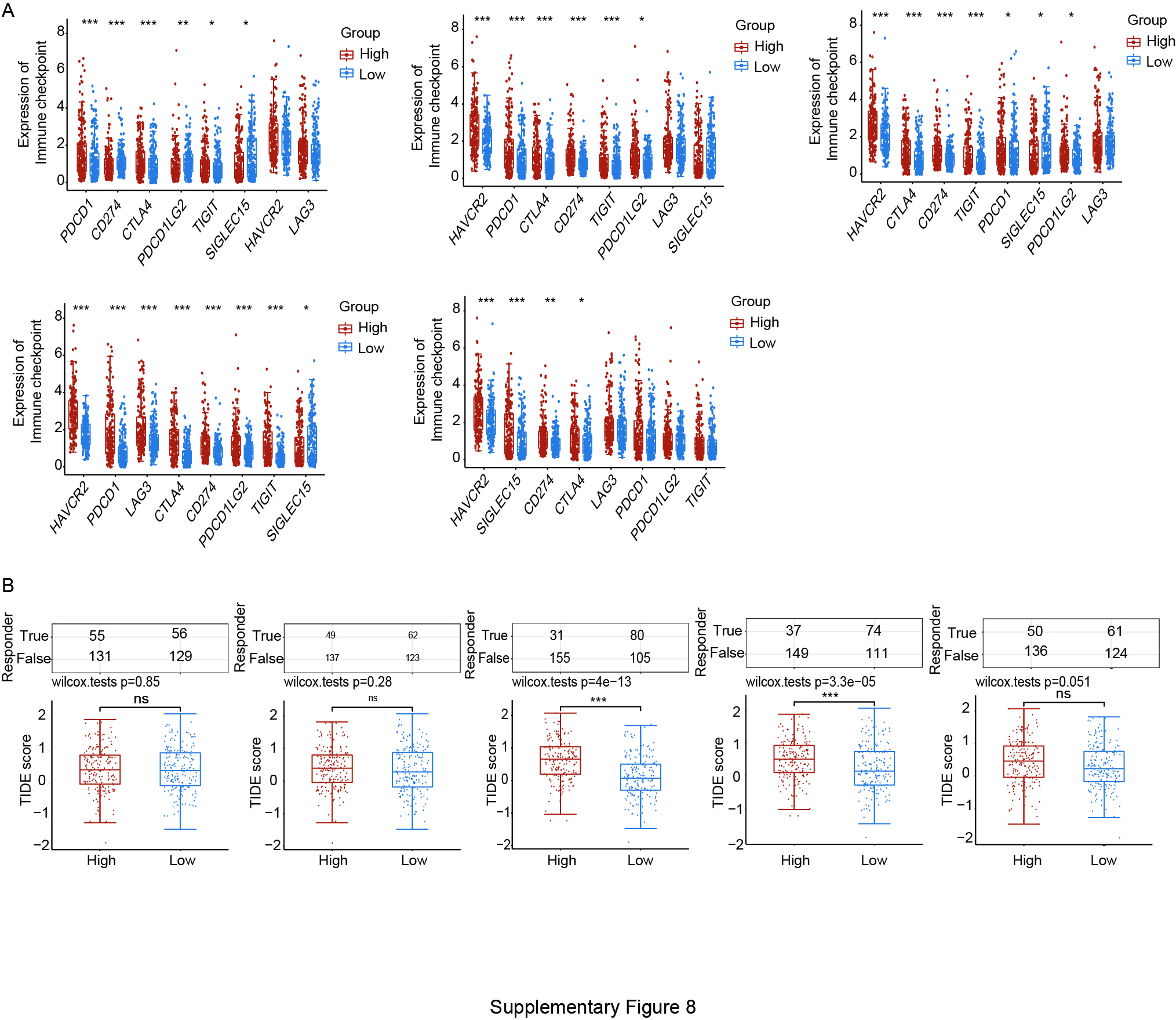

Supplement: Supplementary file 7 [file Image8.JPEG]

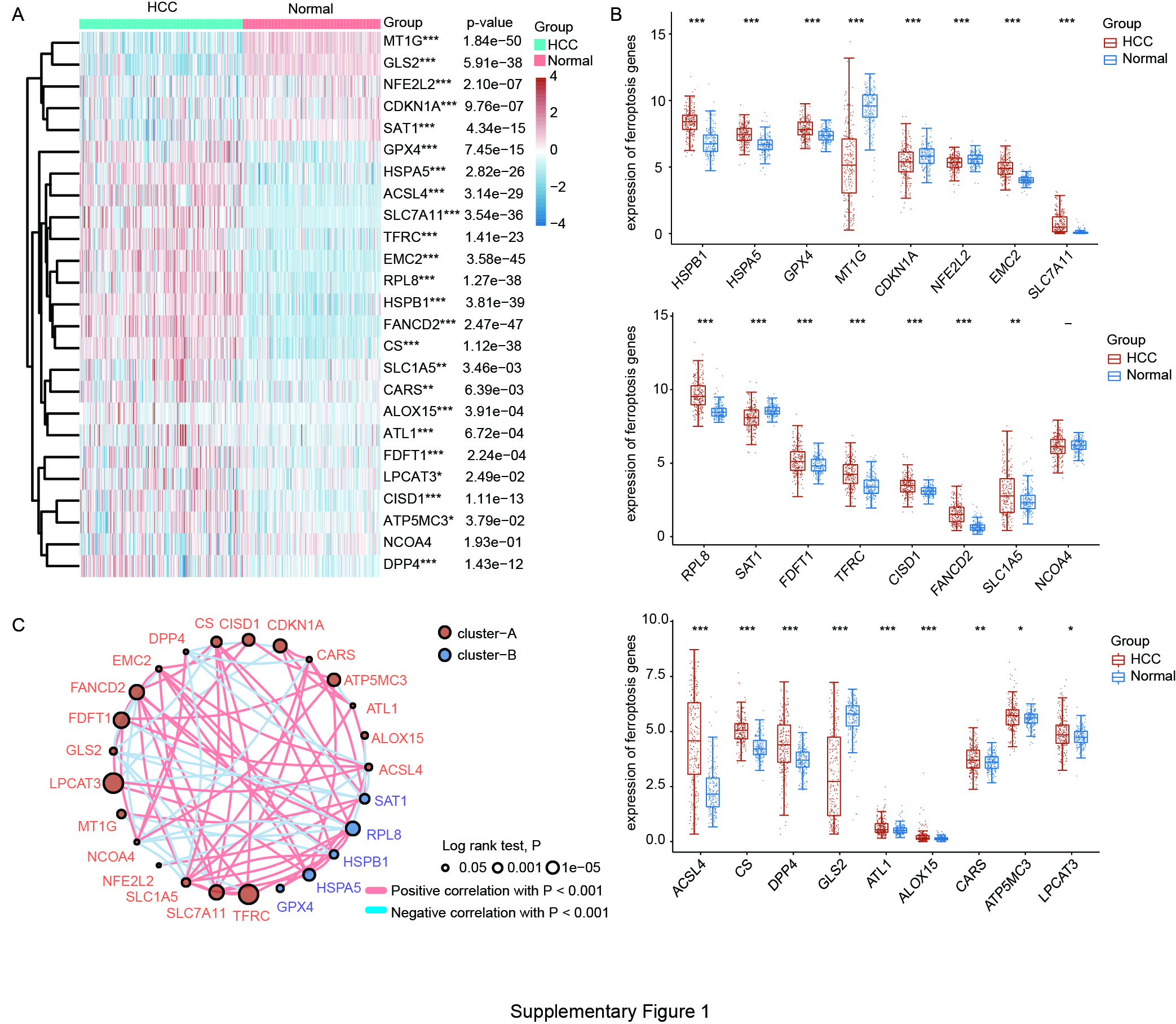

Supplement: Supplementary file 8 [file Image1.jpg]
